# Web-Based Genome-Wide Association Study Identifies Two Novel Loci and a Substantial Genetic Component for Parkinson's Disease

**DOI:** 10.1371/journal.pgen.1002141

**Published:** 2011-06-23

**Authors:** Chuong B. Do, Joyce Y. Tung, Elizabeth Dorfman, Amy K. Kiefer, Emily M. Drabant, Uta Francke, Joanna L. Mountain, Samuel M. Goldman, Caroline M. Tanner, J. William Langston, Anne Wojcicki, Nicholas Eriksson

**Affiliations:** 1 23andMe, Mountain View, California, United States of America; 2 Parkinson's Institute, Sunnyvale, California, United States of America; Georgia Institute of Technology, United States of America

## Abstract

Although the causes of Parkinson's disease (PD) are thought to be primarily environmental, recent studies suggest that a number of genes influence susceptibility. Using targeted case recruitment and online survey instruments, we conducted the largest case-control genome-wide association study (GWAS) of PD based on a single collection of individuals to date (3,426 cases and 29,624 controls). We discovered two novel, genome-wide significant associations with PD–rs6812193 near *SCARB2* (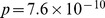, 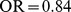) and rs11868035 near *SREBF1*/*RAI1* (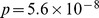, 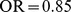)—both replicated in an independent cohort. We also replicated 20 previously discovered genetic associations (including *LRRK2*, *GBA*, *SNCA*, *MAPT*, *GAK*, and the *HLA* region), providing support for our novel study design. Relying on a recently proposed method based on genome-wide sharing estimates between distantly related individuals, we estimated the heritability of PD to be at least 0.27. Finally, using sparse regression techniques, we constructed predictive models that account for 6%–7% of the total variance in liability and that suggest the presence of true associations just beyond genome-wide significance, as confirmed through both internal and external cross-validation. These results indicate a substantial, but by no means total, contribution of genetics underlying susceptibility to both early-onset and late-onset PD, suggesting that, despite the novel associations discovered here and elsewhere, the majority of the genetic component for Parkinson's disease remains to be discovered.

## Author Summary

We conducted a large genome-wide association study (GWAS) of Parkinson's disease (PD) with over 3,400 cases and 29,000 controls (the largest single PD GWAS cohort to date). We report two novel genetic associations and replicate a total of twenty previously described associations, showing that there are now many solid genetic factors underlying PD. We also estimate that genetic factors explain at least one-fourth of the variation in PD liability, of which currently discovered factors only explain a small fraction (6%–7%). Together, these results expand the set of genetic factors discovered to date and imply that many more associations remain to be found. Unlike traditional studies, participation in this study took place completely online, using a collection of cases recruited primarily via PD mailing lists and controls derived from the customer base of the personal genetics company 23andMe. Our study thus illustrates the ability of web-based methods for enrollment and data collection to yield new scientific insights into the etiology of disease, and it demonstrates the power and reliability of self-reported data for studying the genetics of Parkinson's disease.

## Introduction

To date, a number of different genetic susceptibility factors have been identified for Parkinson's disease. Autosomal dominant factors involved in PD include mutations in the *SNCA* (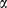-synuclein) [1], [2] and *LRRK2* (leucine-rich repeat kinase 2) [3], [4] genes. Autosomal recessive factors include mutations in the *PARK2* (parkin) [5], *PINK1* (PTEN induced putative kinase 1) [6], *PARK7* (*DJ1*) [7], and *ATP13A2* (ATPase type 13A2) genes [8], [9].

Parkinson's disease is sometimes thought of as consisting of an early-onset form, characterized by familial clustering and high penetrance mutations, and a late-onset form, which occurs more sporadically. In contrast with the evidence for Mendelian inheritance of early-onset PD, studies comparing rates of concordance in monozygotic and dizygotic twins [10]–[12] have traditionally yielded low estimates of heritability for late-onset PD. Evidence of familial aggregation in late-onset PD, when present, is thus sometimes attributed to shared environmental effects or ascertainment bias (see [13], but cf., [14]). Nonetheless, genome-wide association studies have uncovered a few new genes involved in late-onset PD in European [15]–[19] and Japanese [20] populations. These studies have shown repeatedly that common variation in *SNCA* and an inversion of the region containing the *MAPT* (microtubule-associated protein tau) gene are associated with PD. In addition, *NUCKS1* (nuclear casein kinase and cyclin-dependent kinase substrate 1) [16], [20], the *HLA* (human leukocyte antigen) region [18], *GAK* (cyclin G associated kinase) [15], [19], *BST1* (bone marrow stromal cell antigen 1) [20], [21], and five additional loci [22] have recently been associated with PD.

Here, we present a genome-wide association study of PD with a number of distinguishing features. The recruitment of PD cases took place primarily through targeted emails to PD foundations and support groups over the course of 18 months. The large set of controls was recruited through the 23andMe customer database. Determination of case status was conducted through a set of online questionnaires. We present three main scientific results. First, we identify two novel loci associated with PD and replicate many more. Second, we present lower-bound estimates of PD heritability based on observed genome-wide sharing that imply a large fraction of the genetic component underlying the etiology of PD has not yet been discovered. Third, we use sparse regression techniques to construct risk estimation algorithms that account for roughly 6–7% of the total variance in liability to PD and that suggest the existence of true associations lying just beyond genome-wide significance.

## Results

Participants were drawn from the customer database of the personal genetics company, 23andMe, Inc. The majority of cases were recruited into that database through PD support groups and tertiary clinics. All cases reported via web-based surveys having been diagnosed with Parkinson's disease by a physician. Most cases (approximately 84%) also provided detailed information about their disease progression, other diagnoses, symptoms, response to medication, and family history. All participants included in this study were unrelated individuals of primarily European ancestry, based on the criteria described in Materials and Methods.

We used two different sources of data for validating SNPs discovered and models constructed using the 23andMe cohort. To replicate our association results, we exchanged 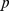-values with the International Parkinson Disease Genomics Consortium (IPDGC), whose dataset consisted of 6,584 cases and 15,470 controls compiled from seven separate cohorts containing individuals of European descent [23]. To validate our risk prediction methods, we used data from the National Institute of Neurological Disease and Stroke (NINDS) Database (see Materials and Methods for details). We did not attempt to replicate our novel associations on the NINDS dataset due to lack of statistical power; similarly, we did not validate our risk prediction methods on the IPDGC cohort as we did not have access to the genetic data for this group. Summary statistics describing all three datasets are provided in Table 1.

**Table 1:** Description of cohorts.

Description	Number	Age	% Male	Age of onset
23andMe controls	29624	48.2 (16.0)	58.5%	–
23andMe cases	3426	64.3 (10.6)	60.3%	57.4 (10.7)
IPDGC controls	15470	–	–	–
IPDGC cases	6584	–	–	–
NINDS controls	799	58.6 (16.4)	41.9%	–
NINDS cases	932	66.2 (11.0)	59.8%	58.5 (13.1)

Age is the average current age for the 23andMe cohort and the average age at collection for the NINDS cohort. Standard deviations are given in parentheses.

### Associations

We performed a GWAS using the 23andMe dataset consisting of 3,426 cases and 29,624 controls, controlling for age, sex, genotyping platform, and five principal components. All 23andMe samples were genotyped using a custom Illumina HumanHap 550+ panel, with 522,782 markers passing quality control (see Materials and Methods). Manhattan and q-q plots can be found in Figures S1 and S2. All SNPs with 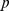-values under 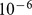 in the 23andMe cohort are shown in Table 2. Summary data for the SNPs in Table 2 can be found in Table S1. All SNPs with 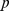-values under 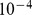 can be found in Table S2. Using a cutoff of 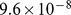 for significance based on a Bonferroni correction across all markers, we identified two novel regions–*SCARB2* and *SREBF1*/*RAI1*–and replicated six previously reported regions–*LRRK2*, *SNCA*, *GBA*, *MAPT*, *MCCC1*/*LAMP3*, and *GAK*. A seventh replication (*SLC41A1*/*PARK16*) and two other potentially novel regions (*RIT2*/*SYT4* and *USP25*) appear nearly genome-wide significant as well.

**Table 2:** GWAS results for all SNPs with 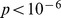 in the 23andMe cohort.

SNP	Chr	Position	Region	Alleles	MAF	Cohort	OR	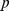
rs34637584	12	39020469	*LRRK2*	G/A	0.002	23andMe	9.615 (6.43–14.37)	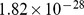
						IPDGC	–	–
i4000416	1	153472258	*GBA*	T/C	0.005	23andMe	4.048 (3.08–5.32)	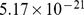
						IPDGC	–	–
rs356220	4	90860363	*SNCA*	C/T	0.375	23andMe	1.285 (1.22–1.36)	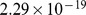
						IPDGC	–	–
rs12185268	17	41279463	*MAPT*	A/G	0.211	23andMe	0.769 (0.72–0.82)	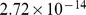
						IPDGC	–	–
rs10513789	3	184242767	*MCCC1*/*LAMP3*	T/G	0.201	23andMe	0.803 (0.75–0.86)	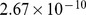
						IPDGC	0.873 (0.83–0.92)	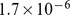
rs6812193	4	77418010	*SCARB2*	C/T	0.365	23andMe	0.839 (0.79–0.89)	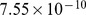
						IPDGC	0.90 (0.86–0.94)	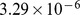
rs6599389	4	929113	*GAK*	G/A	0.075	23andMe	1.311 (1.19–1.44)	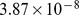
						IPDGC	–	–
rs11868035	17	17655826	*SREBF1*/*RAI1*	G/A	0.309	23andMe	0.851 (0.80–0.90)	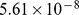
						IPDGC	0.95 (0.91–0.996)	0.033
rs823156	1	204031263	*SLC41A1*	A/G	0.183	23andMe	0.827 (0.77–0.89)	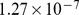
						IPDGC	–	–
rs4130047	18	38932233	*RIT2*/*SYT4*	T/C	0.313	23andMe	1.161 (1.10–1.23)	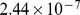
						IPDGC	1.077 (1.03–1.13)	0.0014
rs2823357	21	15836776	*USP25*	G/A	0.376	23andMe	1.149 (1.09–1.21)	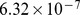
						IPDGC	0.971 (0.93–1.02)	0.187

All genomic positions are given with respect to NCBI build 36.3. Alleles are listed as major/minor and are specified for the forward strand. Odds ratios per copy of the minor allele and 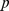-values are provided for the 23andMe cohort and, where requested, the IPDGC replication set. Minor allele frequencies are provided for the 23andMe cohort.

**Figure 1:**
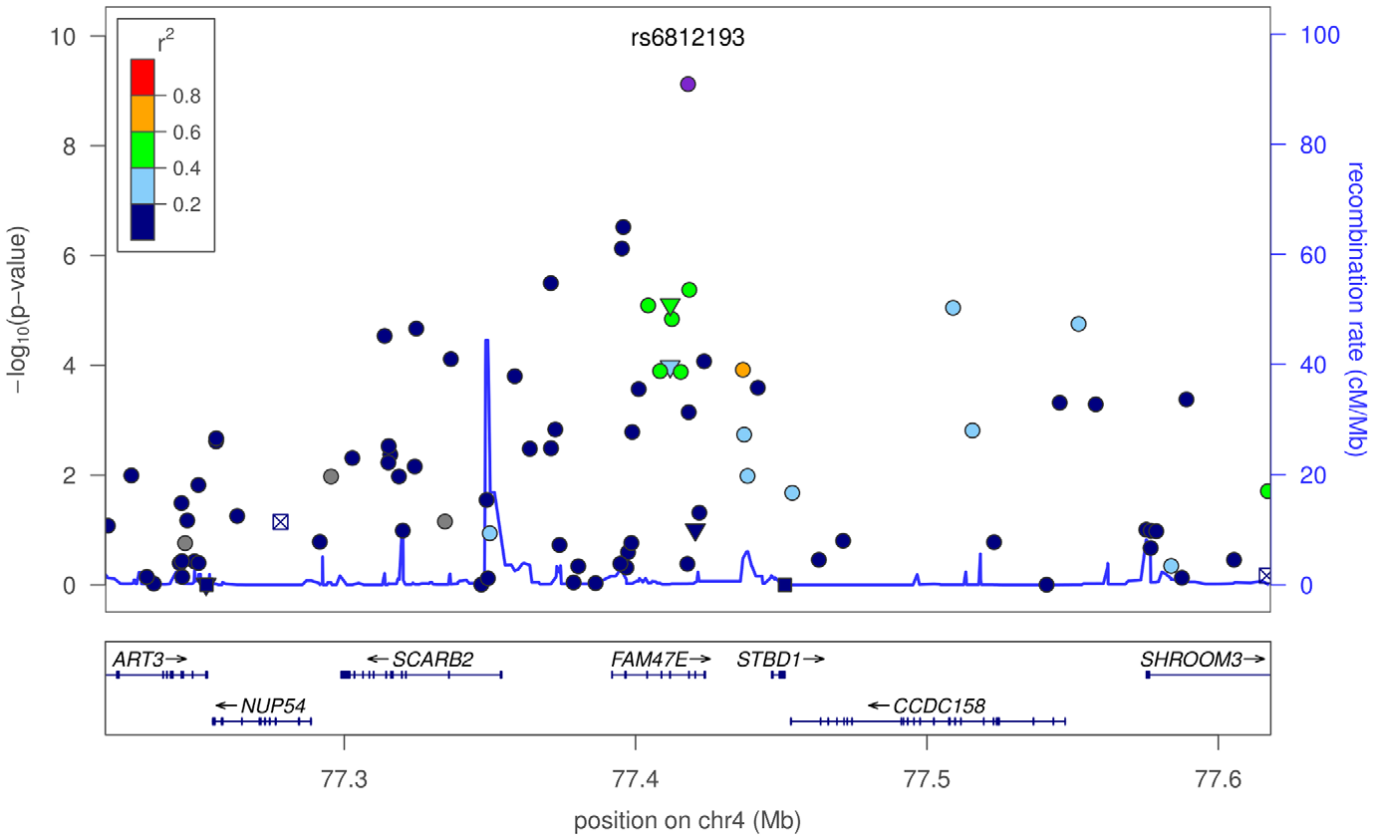
Plot of 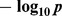-values around rs6812193 and *SCARB2*. In the plot, circles represent unannotated SNPs, upside-down triangles represent non-synonymous variants, and boxes with an “x” are SNPs in regions that are highly conserved across 44 placental mammals. Colors depict the squared correlation (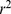) of each SNP with the most associated SNP (i.e., rs6812193). Purple designates the SNP with the strongest association, and gray indicates SNPs for which 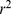 information was missing. Plots were produced using the LocusZoom program [71].

**Figure 2:**
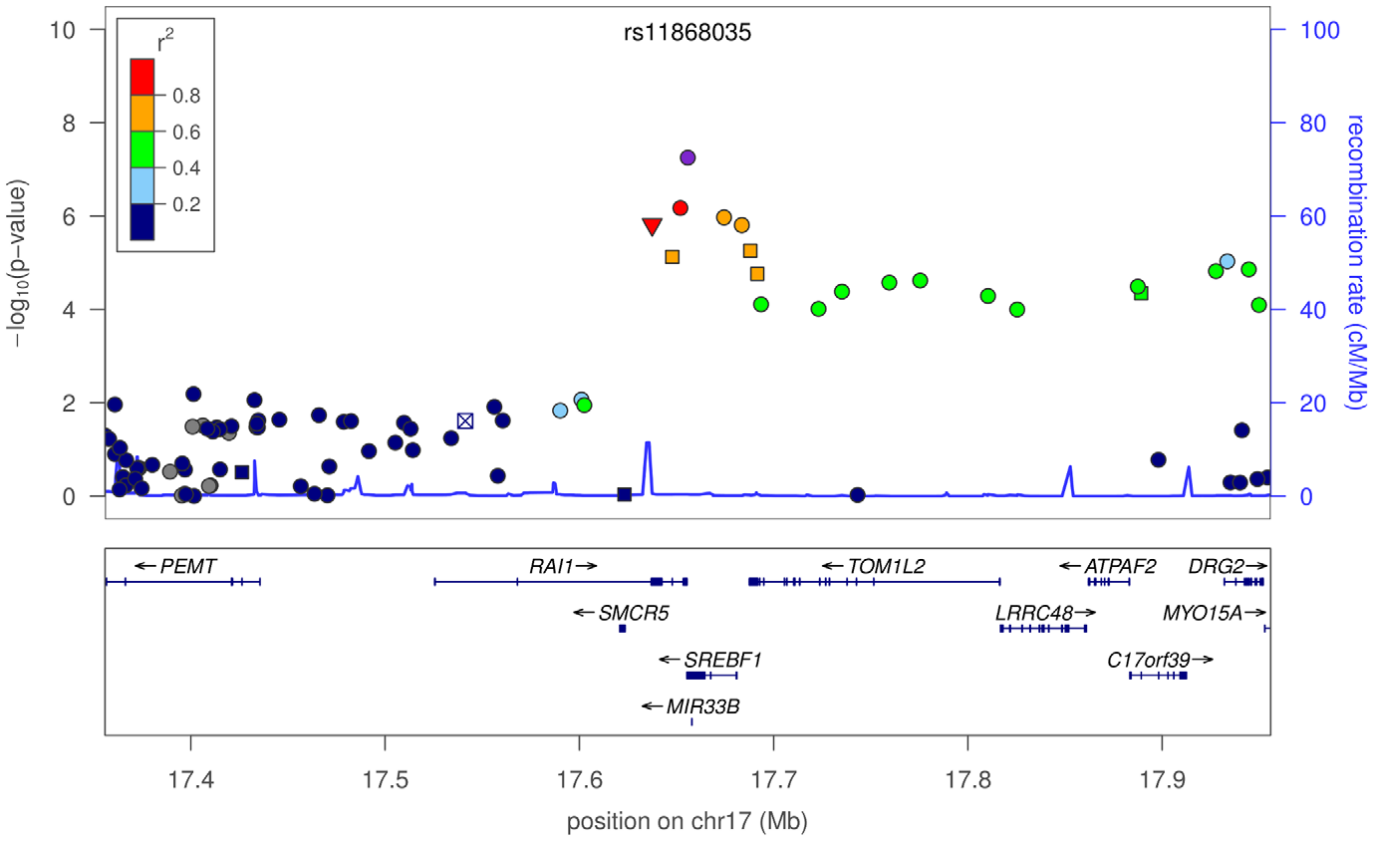
Plot of 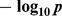-values around rs11868035 and *SREBF1*/*RAI1*. Colors depict the squared correlation (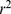) of each SNP with rs11868035. For details, see Figure 1.

The first novel association is rs6812193, with an odds ratio (OR) of 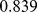 and 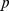-value of 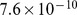, in an intron of *FAM47E* (see Figure 1), upstream of *SCARB2*. This SNP replicates in the IPDGC cohort with an OR of 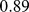 and 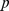-value of 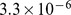. The second novel association is rs11868035, with an OR of 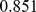 and 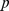-value of 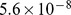, located in an intron of *SREBF1* (see Figure 2). This association also replicates in the IPDGC cohort with an OR of 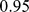 and 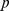-value of 0.03. Of potential interest is a non-synonymous variant (proline to threonine change), with a 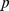-value of 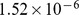, in *RAI1*, rs11649804, in tight linkage disequilibrium (LD) with rs11868035 (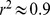).

Among replications of previously reported associations, rs34637584 is the non-synonymous G2019S mutation in the *LRRK2* gene, well known to be associated with PD [24]. *GBA* N370S is one of the mutations causing Gaucher's disease and has recently been associated with PD [25], [26]. These two SNPs, both rare variants, were included as part of the custom set of variants used to genotype the 23andMe cohort. The associations with *SNCA*, *MAPT*, *GAK*, and *SLC41A1* have been reported multiple times. Here we provide the first independent confirmation of the association of rs10513789 in the *MCCC1*/*LAMP3* region with PD, as first reported in [22].

Of the three suggestive associations that do not reach genome-wide significance, rs823156 near *SLC41A1*/*PARK16* has been previously reported [20]. The association with rs4130047 (in an intron of *RIT2*, with an OR of 1.16 and 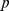-value of 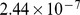) is not quite significant, though it independently appears in the IPDGC cohort with a replication 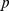-value of 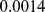, and also is included in the supplement of [15] as a suggestive association under a recessive model. The last suggestive SNP, rs2823357, lies 170 kb upstream of *USP25*, a ubiquitin-specific protease. This association, however, fails to replicate in the IPDGC cohort, with a 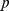-value of 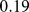. See Figure 5 and Figure 6 in [15] for plots of the *RIT2*/*SYT4* and *USP25* regions.

On the basis of candidate gene studies or modest significance levels in previous GWASs, researchers have proposed associations for many genes with PD to date. We used the set of “Top Results” from the meta-analysis at http://www.pdgene.org/ [27] as well as all SNPs appearing in a PD GWAS with 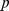-values under 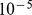 from [28]. After removing SNPs for which we did not have a good proxy, and omitting highly correlated or duplicate SNPs, we were left with 42 potential replications. In addition to *LRRK2* G2019S, 19 other previously reported associations replicated with the correct directionality in the 23andMe cohort using a significance threshold of 0.05 (see Table 3). Of these, 17 of our confidence intervals include the published OR. Of the two that did not, one was using a proxy SNP with a rather weak 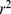 of 0.16, so it is not surprising that our OR is weaker in this case.

**Table 3:** Replication of previously reported associations.

Published SNP (Proxy)	Region	Alleles	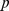	23andMe OR (CI)	Pub. OR (CI)	Pop	Ref.	Grade
*GBA* N370S (i4000416)	*GBA*	T/C	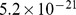	4.048 (3.08–5.32)	3.28 (2.41–4.47)	All	[27]	B
rs356220	*SNCA*	C/T	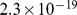	1.285 (1.22–1.36)	1.32 (1.26–1.38)	All	[27]	A
MAPT-H1H2 (rs1876828)	*MAPT*	C/T	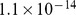	0.764 (0.71–0.82)	0.76 (0.72–0.80)	Euro	[27]	A
rs6812193	*SCARB2*	C/T	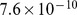	0.839 (0.79–0.89)	0.89 (NR)	Euro	[16]	–
rs823156	*SLC41A1*	A/G	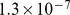	0.827 (0.77–0.89)	0.82 (0.75–0.89)	Asian	[27]	A
rs11711441 (rs11716740)	*LAMP3*	C/T	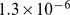	0.821 (0.76–0.89)	0.82 (0.74–0.90)	Euro	[22]	–
rs11248060	*GAK*	C/T	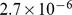	1.202 (1.11–1.30)	1.24 (1.10–1.40)	All	[27]	C
rs2102808 (rs9917256)	*STK39*	G/A	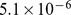	1.199 (1.11–1.30)	1.28 (1.20–1.36)	Euro	[22]	–
rs4698412	*BST1*	A/G	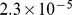	0.891 (0.84–0.94)	0.87 (0.82–0.91)	Asian	[27]	A
rs1491942 (rs11175655)	*LRRK2*	G/A	0.00016	1.167 (1.08–1.26)	1.19 (1.13–1.25)	Asian	[22]	–
rs823128	*NUCKS1*	A/G	0.00019	0.758 (0.65–0.88)	0.70 (0.64–0.76)	Asian	[27]	A
chr1:154105678 (rs10737170)	*SYT11*	A/C	0.00047	1.165 (1.07–1.27)	1.67 (1.50–1.84)	Euro	[22]	–
rs12817488 (rs11060112)	*CCDC62*	A/C	0.0072	0.920 (0.86–0.98)	0.86 (0.82–0.91)	Euro	[22]	–
rs2282048 (rs872606)	*FARP1*	A/C	0.0097	0.932 (0.88–0.98)	0.91 (0.84–0.99)	All	[27]	C
rs12718379	*FGF20*	A/G	0.011	1.072 (1.02–1.13)	1.09 (1.01–1.18)	All	[27]	C
rs7077361	*ITGA8*	T/C	0.0114	0.900 (0.83–0.98)	0.84 (NR)	Euro	[16]	–
rs10200894	2q36.3	C/G	0.0136	0.889 (0.81–0.98)	0.92 (0.83–1.01)	Euro	[27]	C
rs3129882	*HLA*	A/G	0.0194	1.066 (1.01–1.13)	1.16 (1.02–1.32)	All	[27]	C
rs4880	*SOD2*	A/G	0.0304	0.943 (0.89–0.99)	0.88 (0.74–1.04)	Asian	[27]	B
rs797906	*GLIS1*	C/A	0.0578	1.055 (1.00–1.11)	1.08 (1.01–1.15)	All	[27]	C
rs7617877	3p24.1	G/A	0.0859	1.050 (0.99–1.11)	1.23 (1.13–1.33)	Euro	[19]	–
rs6280	*DRD3*	T/C	0.129	0.957 (0.90–1.01)	1.08 (1.02–1.15)	All	[27]	C
rs1079597	*DRD2*	C/T	0.157	1.056 (0.98–1.14)	1.17 (1.00–1.36)	All	[27]	C
rs6710823 (rs4954218)	*AMCSD*	T/G	0.194	1.042 (0.98–1.11)	1.38 (1.29–1.47)	Euro	[22]	–
rs17115100	*CYP17A1*	G/T	0.198	1.061 (0.97–1.16)	0.80 (NR)	Euro	[16]	–
rs7412	*APOE*	C/T	0.2	1.067 (0.97–1.18)	1.15 (1.03–1.28)	All	[27]	C
rs12063142	*TAS1R2*	C/T	0.252	1.035 (0.98–1.10)	NR (NR)	Euro	[17]	–
rs2010795	*PDXK*	G/A	0.277	1.032 (0.98–1.09)	1.09 (1.02–1.16)	All	[27]	C
rs10464059 (rs1862326)	5q35.3	G/T	0.308	0.962 (0.89–1.04)	1.33 (1.19–1.52)	Euro	[17]	–
rs1799836	*MAOB*	T/C	0.414	0.983 (0.94–1.03)	1.10 (1.01–1.20)	All	[27]	C
rs11030104	*BDNF*	A/G	0.485	0.977 (0.92–1.04)	1.12 (1.04–1.22)	All	[27]	C
rs1043424	*PINK1*	A/C	0.492	1.021 (0.96–1.08)	0.91 (0.81–1.01)	Euro	[27]	C
rs1994090	*LRRK2*	T/G	0.496	1.023 (0.96–1.09)	1.39 (1.24–1.56)	Asian	[20]	–
rs1801133	*MTHFR*	G/A	0.506	0.981 (0.93–1.04)	1.12 (1.02–1.22)	Asian	[27]	B
rs1223271	*C20orf82*	G/A	0.553	0.976 (0.90–1.06)	0.85 (NR)	Euro	[16]	–
rs17329669	7p14.2	A/G	0.56	0.978 (0.91–1.05)	1.13 (1.03–1.24)	All	[27]	C
rs12431733	*BMP4*	C/T	0.678	0.989 (0.94–1.04)	1.13 (NR)	Euro	[16]	–
rs5174	*LRP8*	C/T	0.841	0.994 (0.94–1.05)	0.93 (0.87–1.00)	All	[27]	C
rs13312	*USP24*	C/G	0.874	0.995 (0.93–1.06)	0.76 (0.66–0.86)	All	[27]	A
rs1801582	*PARK2*	C/G	0.881	0.995 (0.93–1.07)	0.79 (0.64–0.97)	All	[27]	C
rs1805874 (rs2205108)	*CALB1*	G/T	0.882	1.004 (0.95–1.06)	1.12 (1.01–1.25)	All	[27]	C
rs4837628	*DBC1*	T/C	0.925	0.997 (0.95–1.05)	0.79 (0.72–0.87)	Euro	[17]	–

SNPs were taken from the PDGene “Top Results” list of meta-analyses [27] and the NHGRI list of associations [57]. Alleles are given with respect to the forward genomic strand for NCBI build 36.3 and are listed as major/minor. Where a proxy SNP was used, alleles refer to the proxy SNP. Published OR is the published odds ratio per copy of the minor allele for the association, as reported in the corresponding reference. Overall grades for SNPs based on the Venice criteria [72] were taken from the PDGene list, where available, and omitted otherwise. “NR” means that an OR or CI was not reported. 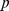-values and power calculations were calculated assuming a two-sided test. Our power to replicate a given association conditional on the published odds ratio and the minor allele frequency using a 0.05 threshold for significance was above 86% for all associations except for rs10200894, for which it was 59%. “Pop” refers to the ancestry in which the association was discovered, as taken from the PDGene list or the original paper where applicable. “All” indicates that multiple studies were used in the PDGene meta-analysis, irrespective of ancestry.

We had good power (over 86% in all but one case) to replicate all 42 of these associations, assuming the reported odds ratio (from the meta-analysis in the case of associations from [27], from the original paper otherwise) was correct. Thus the failure to replicate many of the reported associations (including two with “A” or “B” meta-analysis grades) is likely partially due to inflation of the odds ratios in those reports, although a few of the associations that were discovered in Asian populations, such as rs1994090, may simply not have the same effect in European populations.

Finally, we note that in all analyses above, we restricted consideration to a log-additive model of association. Allowance for dominant and recessive models might lead to increased power for detection in cases where a log-additive model is not appropriate, and may also affect the success rates in our replication experiment.

### Heritability

To characterize the extent to which genetic factors tagged on genotyping panels play a role in PD susceptibility, we applied a recently proposed approach for estimating heritability based on genome-wide sharing between distantly related individuals [29], [30]. Using the 23andMe cohort, we estimated the heritability of PD to be 0.272 with 95% CI of 0.229 to 0.315. This estimate refers only to the proportion of phenotypic variance arising from causal variants that are in LD with the SNPs on our genotyping platform, which may be less than the corresponding proportion for all causal variants. This estimate has only a mild dependence on the assumed prevalence: using prevalences of 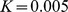 or 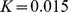 gives estimates of 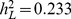 or 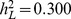, respectively. Thus, our estimates generally suggest a lower-bound on the actual heritability in the 0.25 to 0.3 range.

Table 4 compares our heritability estimates using the 23andMe cohort with an estimate computed for the NINDS cohort based on the same analytic methods, and with numbers obtained from the literature. Where only relative recurrence risk ratios were provided, we inferred the corresponding heritability of liability under an assumption of no shared environmental covariance [31]; in practice, such an assumption is unlikely to hold for close relatives, and as such, the estimates of heritability we have inferred from those studies are likely to be upwardly biased. We note that our estimates of heritability for PD are most consistent with estimates from prior twin studies, though with substantially tighter confidence intervals even after accounting for the uncertainty in prevalence.

**Table 4:** Heritability estimates.

Source	Description	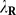	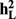	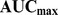	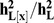
NINDS	All PD	-	0.229 (0.000–0.543)	0.833 (0.500–0.935)	0.077 (0.036- 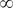 )
23andMe	All PD	-	0.272 (0.229–0.315)	0.857 (0.833–0.877)	0.065 (0.056–0.077)
23andMe	Early-onset PD ( 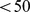 )	-	0.306 (0.136–0.476)	0.873 (0.766–0.933)	0.057 (0.037–0.129)
23andMe	Late-onset PD ( 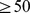 )	-	0.285 (0.224–0.346)	0.863 (0.830–0.890)	0.062 (0.051–0.078)
[12]	Twin study; broad-definition PD	-	0.30 (0.00–0.47)	0.87 (0.50–0.93)	0.059 (0.037- 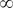 )
[10]	Twin study; all PD	-	0.274 (0.000–0.708)	0.858 (0.000–0.976)	0.064 (0.025- 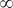 )
[10]	Early-onset PD ( 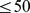 )	-	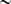 1.0 (0.33–1.00)	0.996 (0.884–0.996)	0.018 (0.018–0.053)
[10]	Late-onset PD ( 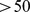 )	-	0.068 (0.00–0.59)	0.693 (0.000–0.958)	0.258 (0.030- 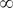 )
[48]	Family study; all PD	-	0.401	0.910	0.044
[48]	Early-onset PD ( 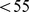 )	-	0.169	0.793	0.104
[48]	Late-onset PD ( 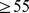 )	-	0.453	0.926	0.039
[73]	Family study; all PD	-	0.60 (0.40–0.80)	0.96 (0.91–0.99)	0.029 (0.022–0.044)
[14]	Offsprings; all PD	3.0	0.35	0.89	0.050
[14]	Late-onset PD ( 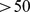 )	3.2	0.38	0.90	0.046
[74]	Parents and siblings; all PD	3.92	0.456	0.927	0.038
[74]	Early-onset PD ( 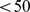 )	7.76	0.747	0.980	0.024
[74]	Late-onset PD ( 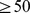 )	2.95	0.348	0.891	0.050

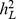
 denotes the heritability of liability for PD, with a 95% CI provided where available. In the case of [10], confidence intervals were estimated via a bootstrap procedure based on numbers provided in the original paper. For studies that did not provide direct estimates of heritability, the relative recurrence risk ratio 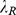 was used to estimate 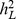 under the assumption of no shared environmental covariance (see Materials and Methods). 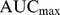 denotes the maximum theoretical AUC corresponding to the given heritability of liability, assuming a disease prevalence of 0.01. 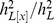 denotes the proportion of additive genetic variance explained by a genetic profile that achieves an AUC of 0.6 (see Materials and Methods).

### Risk prediction

Given our estimates of the genetic contribution to PD, we then sought to determine the proportion of this contribution that we could attribute to specific genetic factors on our genotyping panel by constructing risk prediction models for PD. We considered two settings: an internal five-fold cross-validation experiment, where we divided the 23andMe cohort into five matched sets of cases and controls and computed predictions for each set using models trained using the other four sets; and an external cross-validation experiment, where we trained risk prediction models using the entire 23andMe cohort and tested them on the NINDS cohort after restricting both datasets to the set of common SNPs passing quality control for each dataset individually.

In both the internal and external validation experiments, we measured the discriminative accuracy of the risk prediction algorithm using the area under the receiver operating characteristic curve (AUC). The AUC for a model can be interpreted as the probability that a randomly selected case will have a higher estimated risk of developing PD than a randomly selected control. An AUC of 1 implies that a model discriminates perfectly between cases and controls, whereas an AUC of 0.5 corresponds to a model based on random guessing. To measure predictive accuracy, we used a covariate-adjusted measure of AUC that removed the effect of potential confounding by sex, age, population structure, and, where appropriate, cross-validation fold or genotyping platform (see Materials and Methods).

To avoid making manual decisions in the choice of SNPs to include, we used a sparse logistic regression algorithm for building risk prediction models, and varied the strength of the sparsity-inducing prior (see Materials and Methods). For a given training set, this procedure generated a series of risk prediction models of differing size, each of which we characterized using an approximate theoretical upper bound on the expected number of false positive SNPs, 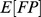; here, 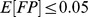 corresponds to a model containing only genome-wide significant associations. In the internal five-fold cross-validation experiment (see Table 5), the differences in AUCs between each of the largest three models (i.e., 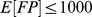, 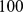, or 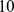) and each of the smallest two models (i.e., 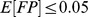 or 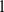) were significant (e.g., 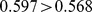 comparing the largest and smallest models, one-sided 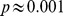; see Table S7). In the external cross-validation results, the four largest models were significantly better than the genome-wide significant model (e.g., 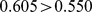 comparing the largest and smallest models, one-sided 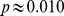).

**Table 5:** Internal and external cross-validation experiments using sparse logistic regression.

	Internal Validation			External Validation		
Signif. Threshold	Avg SNPs	Avg Regions	AUC	SNPs	Regions	AUC
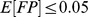	9.0	6.6	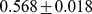	11	9	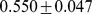
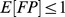	18.4	15.4	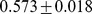	22	19	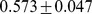
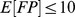	41.6	35.0	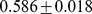	60	51	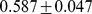
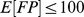	156.0	138.8	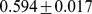	220	195	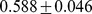
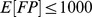	698.4	639.2	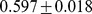	803	727	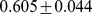

The internal five-fold cross-validation experiment was performed using only the 23andMe cohort. The external cross-validation experiment was performed by training on the 23andMe cohort and testing on the NINDS cohort. “SNPs” denotes the number of SNPs included in the fitted model. “Regions” denotes the number of distinct LD blocks represented by the SNPs in the fitted model. Each AUC value represents a covariate-adjusted AUC. For the internal validation experiment, average values are provided for SNPs and Regions, providing an average over all five cross-validation folds, and AUCs were computed by pooling predictions over the five cross-validation folds. For each row of the table, the sparsity inducing prior was chosen to achieve the approximate upper bound on the expected false positive rate indicated in the first column; here, 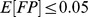 corresponds to a model containing only genome-wide significant associations, whereas 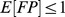 corresponds to suggestive associations. In each of the internal and external validation experiments, models with AUCs in bold are significantly better than non-bold models (see Table S3).

Taken at face value, these results seem to suggest that the larger models, which include many more genomic regions than those deemed genome-wide significant, may harbor associations that account for their increased predictive accuracy. An alternative possibility, however, is that the differences in performance between models are actually just a consequence of differing levels of bias arising from the use of sparsity-inducing regularization. In Text S1 and Table S4, we present an argument using a bias-corrected version of our external cross-validation experiment that the above caveats do not explain the improved accuracy for the 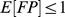 and 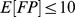 models, thus suggesting that these models are likely to harbor additional important loci for PD; see Tables S5 and S6 for the SNPs included in these models. We note that this argument may not be the only way of demonstrating the existence of meaningful associations beyond the genome-wide significance threshold; closely related arguments based on the sparse regression methods [32] or genetic profile scores have also been previously proposed [33].

Regardless, our heritability estimates imply an upper bound on AUC for a genetic risk prediction model of roughly 0.83 to 0.88 based on the method of [31], though this would rise if the actual heritability were higher. Based on these numbers, the genetic risk prediction models detailed previously, which attain an AUC of roughly 0.6 in our cross-validated tests, account for approximately 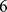
–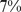 of the total genetic variance in liability (see Materials and Methods).

## Discussion

We found two novel associations at a genome-wide level of significance near *SCARB2* (rs6812193) and *SREBF1*/*RAI1* (rs11868035), both of which were replicated in data from [23]. We also report two novel associations (near *RIT2* and *USP25*) just under the level of significance, one of which (*RIT2*) was also replicated. While it is difficult to pinpoint any causal genes from a GWAS, there are a few biologically plausible candidates worthy of discussion.

The PD-associated SNP rs6812193 lies in an intron of the *FAM47E* gene, which gives rise to multiple alternatively spliced transcripts, many of which are protein-coding; the functions of these hypothetical proteins are unknown. A more attractive candidate, located 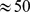 kb centromeric to the SNP, is *SCARB2* (scavenger receptor class B, member 2), which encodes the lysosomal integral membrane protein type 2 (LIMP-2). LIMP-2 deficiency causes the autosomal-recessive disorder Action Myoclonus-Renal Failure syndrome (AMRF), which combines renal glomerulosclerosis with progressive myoclonus epilepsy associated with storage material in the brain [34]. LIMP-2 is involved in directing 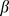-glucocerebrosidase to the lysosome where it hydrolyzes the 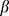-glycosyl linkage of glucosylceramide [35]. Deficiency of this enzyme due to mutations in its gene (*GBA*) causes the most common lysosomal storage disorder, Gaucher's disease. Recently, mutations in *GBA* have also been identified in PD [36], pointing to a possible functional link between the newly identified candidate gene *SCARB2* and PD.

rs11868035 appears in an intron of the alternatively spliced gene, *SREBF1* (sterol regulatory element-binding transcription factor 1), within the Smith-Magenis syndrome (SMS) deletion region on 17p11.2. *SREBF1* encodes SREBP-1 (sterol regulatory element-binding protein 1), a transcriptional activator required for lipid homeostasis, which regulates cholesterol synthesis and its cellular uptake from plasma LDL [37]. Studies of neuronal cell cultures have implicated SREBP-1 as a mediator of NMDA-induced excitotoxicity [38]. rs11868035 is directly adjacent to the acceptor splice site for the C-terminal exon of the SREBP-1c isoform of the protein [39], suggesting that the effect of the polymorphism may be specifically related to the splicing machinery for this protein. The mutation is also in strong LD with rs11649804, a nonsynonymous variant in the nearby gene *RAI1* (retinoic acid-induced protein 1), which regulates transcription by remodeling chromatin and interacting with the basic transcriptional machinery. Heterozygous mutations in *RAI1* reproduce the major symptoms of SMS, such as developmental and growth delay, self-injurious behaviors, sleep disturbance, and distinct craniofacial and skeletal anomalies [40]. Future work is needed to identify the functionally important variant(s) responsible for this association.

The SNP rs4130047, slightly below the genome-wide significance threshold, lies in an intron of the *RIT2* (Ras-like without CAAX 2) gene that encodes Rit2, a member of the Ras superfamily of small GTPases. Though we do not claim this SNP as a confirmed replication, there are a number of reasons to suspect that this association may also be real. Rit2 binds calmodulin in a calcium-dependent manner, and is thought to regulate signaling pathways and cellular processes distinct from those controlled by Ras [41]. It localizes to both the nucleus and the cytoplasm. Independent of our study, *RIT2* was previously proposed as a candidate gene for PD, based on the possibility that dopaminergic neurons may be especially vulnerable to high intracellular calcium levels, perhaps through an interaction with 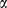-synuclein [42]. The PD-associated region contains another biologically plausible candidate gene, *SYT4* (synaptotagmin IV), which encodes synaptotagmin-4, an integral membrane protein of synaptic vesicles thought to serve as 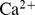 sensor in the process of vesicular trafficking and exocytosis. It is expressed widely in the brain but not in extraneural tissues [43]. Homozygous Syt4−/− mouse mutants have impaired motor coordination [44]. *SYT4* is particularly interesting as a SNP near *SYT11* (synaptotagmin XI) has been associated with PD in [22], and the encoded protein, synaptotagmin-11, is known to interact with parkin [45].

The suggestively associated SNP rs28233572 lies in a gene-poor region with only one candidate gene downstream, *USP25*, encoding ubiquitin specific peptidase 25, which regulates intracellular protein breakdown by disassembly of the polyubiquitin chains. Other ubiquitin-specific proteases (*USP24*, *USP40*) have been proposed as candidate genes for PD [46] (although *USP24* fails to replicate here, see Table 3).

Our heritability estimates, which suggest that genetic factors account for at least one-fourth of the total variation in liability to PD, represent the tightest confidence bounds determined for the heritability of PD to date. These estimates, which rely on observed genetic sharing rather than predicted relationship coefficients, avoid confounding from shared environmental covariance by restricting attention to very distantly related individuals. Furthermore, they complement estimates of heritability from twin studies by considering large numbers of individuals with low amounts of genetic sharing, rather than small numbers of twin pairs with large amounts of genetic sharing.

These estimates should only be interpreted as lower bounds on the actual heritability of liability of PD for two reasons. First, they only reflect phenotypic variation due to causal variants in LD with SNPs on the genotyping platform. Second, they only capture the contribution to additive variance that arises from a polygenic model of many SNPs of small effect, but do not include the variance arising from known specific associations. This limitation is most apparent in our estimate of heritability based on only early-onset cases (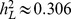), which is considerably lower than reported in prior twin studies (e.g., 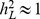 in [10]). In early-onset PD, mutations in six specific genes (*SNCA*, *PRKN*, *PINK1*, *DJ1*, *LRRK2*, and *GBA*) have been reported to account for 16% of cases [47]; these specific mutations are not directly accounted for in our estimate, which is based on a polygenic model. We note that a similar effect may explain the low heritability estimate for early-onset PD in [48]. Thus, the actual heritability of PD, and the corresponding true upper bound on discriminative accuracy achievable through genetic factors, may be even higher than the estimates we provide.

Our estimates also indicate a substantial genetic component for late-onset PD (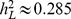), for which previous estimates of heritability have been inconclusive due to the lack of statistical power (e.g., 0.068 in [10] and 0.453 in [48]). One might ask, if late-onset PD is indeed so heritable, why do cases frequently appear sporadically in the general population? Following the analysis of [49], if one were to assume a heritability of 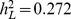 and an average of three children per family, then the proportion of sporadic cases (i.e., no parent, child, sibling, grandparent, aunt or uncle, or first cousin with PD) among all PD cases would be 64% for a prevalence of 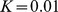; in the 23andMe cohort, 69% of PD cases would be considered sporadic by this definition based on self-reported family history. Similarly, the expected proportion of PD cases with no affected parent or sibling would be 88% under the same assumptions, compared with 84% as reported in [50], or 89% based on the cohort in [51]. These examples illustrate the fact that the presence or absence of a familial pattern cannot always be used to determine pathogenesis, especially for diseases that are rare and have a complex etiology.

Overall, our risk prediction results are consistent with a measured AUC of roughly 0.6. The cross-validated AUCs presented here should be distinguished from more usual measurements of AUC in genome-wide association studies, which are typically only estimated on the development set, and which rely on weighted combinations of SNPs with independently estimated odds ratios. In some cases, the bias resulting from lack of proper external validation can be quite large. For example, a simple genetic profile score based on multiplying together odds ratios for the SNPs in Table 2 appears to achieve an AUC of 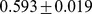 in the 23andMe data (or 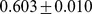 if no covariate adjustment is performed) making it appear competitive with some of the best models described in Table 5. However, when the same model is evaluated in the NINDS data, the AUC drops to 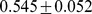, exhibiting a drop in performance characteristic of models that have been overfit to their training data. In contrast, the consistency between the internal and external validation results in the models shown in Table 5 demonstrate not only the predictiveness of our models within the 23andMe cohort but also their ability to generalize to other populations.

Our empirical demonstration that including SNPs beyond the genome-wide significant level provides improved discriminative power mirrors the recent results of [32], which also studied the performance of sparse regression methods in a risk prediction setting. In an applied setting where the goal is to achieve the best predictive accuracy rather than to isolate the contribution of individual genetic factors, however, even higher discriminative accuracies may be possible if one were to incorporate these covariates as part of the predictive models. Even without these, however, significant improvements in risk prediction are likely still possible, with our heritability analyses indicating asymptotic target AUCs above 0.8.

Our AUCs are generally conservative for a number of reasons. In the internal experiments, they were obtained by training on only 80% of the data. In the external experiments, the models included only the SNPs in common between the 23andMe and NINDS datasets and thus excluded several SNPs with large effects in *LRRK2* and *GBA* that may add a percent or more to the AUC if included. Furthermore, our analyses adjusted for confounding from population structure and other covariates so as to ensure that the discriminative accuracies we reported were specifically due to genetic effects.

Finally, we note that data for the 23andMe cohort used in this study were acquired in a novel manner, using genotype and survey data acquired through a commercial online personal genetic testing service. The use of self-reported phenotype data raised some unique challenges. For example, our cohort was not a true population sample for a number of reasons, such as the general bias toward higher socioeconomic status, as typical of 23andMe customers. In general, however, we would not expect these ascertainment biases to substantially affect our conclusions unless their effects varied differentially between the case and control sets.

As another example, in compiling the cohort, we used participants with varying levels of completeness in their self-reported data (see Materials and Methods). Out of the 3,426 cases in the 23andMe cohort, though most cases reported having PD in a questionnaire, 482 affirmatively stated they had PD upon entry to the research study but did not fill out any PD-related questionnaire during the study. However, we did not see a large difference between those answering questions and not. Among the 11 associations presented in Table 2, only the association with *MAPT* showed a significant difference between the cohort who answered a questionnaire and those who did not (see Table S7). Also, approximately 84% of the cases filled out a questionnaire, and of them, over 96% reported a PD diagnosis. Even if a larger fraction (say 10–15%) of those who did not take a questionnaire did not have PD, the gain in power from the additional cases would more than offset the loss of power from having some 50 more false positive cases.

Despite the challenges associated with using self-reported data collected through online surveys, ultimately, our results lend credibility to the accuracy of this novel research design. For example, the agreement between our study and previous studies in terms of the ORs estimated for the 19 associations replicated in Table 3 strongly suggests that our cohort is similar to those used in other PD studies. Similarly, the consistency of AUCs and heritability estimates across our cohort and the NINDS cohort both suggest a limited role of bias in our study.

Importantly, our mode of data collection also provided a number of clear benefits. The use of internet-based techniques enabled rapid recruitment of a large patient community. The 3,426 cases in this study were enrolled in about 18 months, with over half joining in the first month of the study. Also adding significantly to the power and robustness of this study was the availability of a large cohort of controls derived from the 23andMe customer base. By using a non-traditional recruitment approach, we thus were able to attain good power for our study through large sample sizes. To our knowledge, this study represents the largest genome-wide association study of Parkinson's disease conducted on a single cohort to date, with only a recent meta-analysis achieving a larger number of cases [22]. We suggest that this methodology for study design may prove advantageous for other conditions where the advantage of having a large cohort is paramount for detecting subtle genetic effects.

In summary, we have for the first time used a rapid, web-based enrollment method to assemble a large population for a genome-wide association study of PD. We have replicated results from numerous previous studies, providing support for the utility of our study design. We have also identified two new associations, both in genes related to pathways that have been previously implicated in the pathogenesis of PD. Using cross-validation, we have provided evidence that many suggestive associations in our data may also play an important role. Using recently developed analytic approaches developed for GWAS that take into account the ascertainment bias inherent in a case-control population, we have estimated the genetic contribution to PD in this sample. These findings confirm the hypothesis that PD is a complex disorder, with both genetic and environmental determinants. Future investigations, expanded to include environmental as well as genetic factors, will likely further refine our understanding of the pathogensis of PD, and, ultimately, lead to new approaches to treatment.

## Materials and Methods

### Study populations

The 23andMe cohort consisted of customers of 23andMe, Inc., a personal genetics company. Patients with PD were recruited to join this cohort through a targeted email campaign in conjunction with the Michael J. Fox Foundation, The Parkinson's Institute and Clinical Center, and many other PD patient groups and clinics. Emails or hard copy mailings were sent to all individuals who had registered with these groups as PD patients. A limited number of patients were also recruited in person at PD workshops and conferences. Family members of individuals with the *LRRK2* G2019S were also recruited to participate in the general Parkinson's disease research at 23andMe, without regard to Parkinson's disease status; however, most of these individuals were not included in the cohort used for this particular study, due to our restriction of the dataset to unrelated individuals (see below). Patients were invited to fill out a screening questionnaire asking if they had been diagnosed with PD and their physician's name, phone number, and institution. Patients who stated they had been diagnosed with PD and who gave complete, non-suspicious answers to the other questions were offered the 23andMe Personal Genome Service for a nominal fee of $25.

Individuals included in the 23andMe cohort were selected for being of primarily European ancestry, as determined through an analysis of local ancestry via comparison to the three HapMap 2 populations, using an unpublished method substantially similar to [52]. A maximal set of unrelated individuals in the 23andMe cohort was chosen for the analysis using a segmental identity-by-descent (IBD) estimation algorithm (as used in [53]). Individuals were defined as related if they shared more than 700 cM IBD, including both regions where the two individuals share either one or both genomic segments identical-by-descent. This level of relatedness (roughly 20% of the genome) corresponds approximately to the minimal expected sharing between first-cousins in an outbred population. We determined that 29 individuals were included in both the 23andMe and NINDS cohorts and hence were removed from the latter cohort for all analyses.

Genotype and phenotype data for the National Institute of Neurological Disease and Stroke (NINDS) cohort were obtained from the NINDS Database found at http://www.ncbi.nlm.nih.gov/gap through dbGaP accession number phs000089.v3.p2, supplemented with individual-level data for 200 subjects from phs000089.v2.p2 who were left out of the later version as of December 12, 2010. Cases in the NINDS cohort consisted of North American Caucasians with Parkinson's disease, as assessed by a neurologist. Controls consisted of neurologically normal, unrelated, white individuals with no family history for a number of neurological conditions, including Parkinson's disease. A complete description of the inclusion and exclusion criteria can be found directly at the NINDS Database website as referenced above, and in related studies using the data from this cohort [16], [54], [55].

This study was conducted according to the principles expressed in the Declaration of Helsinki. The 23andMe study protocol and consent were approved by the external AAHRPP-accredited IRB, Ethical and Independent Review Services (E&I Review). Our consent and privacy statement preclude the sharing of individual-level data without explicit consent. We have, however, shared summary statistics for all SNPs with 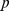-values under 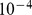 (Table S2). We also hope to further collaborate with the scientific community using this data. The NINDS dataset was analyzed anonymously.

### Self-reported diagnosis

Patients recruited through the PD outreach initiative as well as individuals from the general 23andMe customer base were asked to take online questionnaires, including a general medical questionnaire and a detailed questionnaire specifically on PD (covering disease onset, diagnosis, and symptoms). Both of these questionnaires asked the subject if he or she had ever received a PD diagnosis from a physician and if so, the age of onset. The detailed questionnaire also asked for much more specific information regarding the symptoms, clinical history, and family history of the patient.

We selected as cases all participants who provided an affirmative diagnosis of PD from a physician (on the initial screening form or on either of the two questionnaires) and who did not provide any potentially contradictory information, defined here as:

- Providing an affirmative answer to the screening question but only negative answers to the two questions about PD.
- Answering “yes” to PD on the general medical questionnaire but “no” to PD on the detailed questionnaire.
- Stating their diagnosis changed because they no longer had symptoms or because the cause of their symptoms was unknown.
- Stating they had been diagnosed with any of 19 other neurological conditions (see Table S8).

Due to the low prevalence of PD, we used controls taken from general 23andMe customer base in the analysis. Some of the controls filled out no questionnaires; however, others answered questions about possible PD-like symptoms or filled out a general medical history. In order to maximize our power to detect genetic associations with PD, we excluded some putative controls who might be at higher probability for developing PD in the future. Thus, the controls consisted of all consented European 23andMe customers who met all of the following criteria:

- Were not part of a PD related recruitment drive (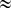
130 individuals excluded)
- Did not report a diagnosis of PD, Parkinsonism, dementia, cognitive impairment, senility, tremor disorder, Alzheimer's disease or memory loss (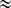
240 individuals excluded)
- Did not report a family history of PD (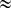
780 individuals excluded)
- Reported a maximum of two of the following PD-like symptoms (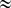
280 individuals excluded):
  Trembling or shaking of any body part
  Handwriting became slower, smaller, or shakier (each considered a separate symptom)
  Speech or voice become softer
  Dragging one or both feet while walking
  Feet shuffling while walking
  Walking more slowly
  Taking smaller steps than before
  Steps becoming faster and faster
  Feet getting stuck as if glued to the floor
  Swinging arms less than before
  Stooping or bending forward more than before
  Falling or balance trouble

Approximately 1,430 individuals in total were excluded from the control set due to these filters. We note that as a consequence of both our exclusion criteria and other recruitment biases associated with the 23andMe customer base, our controls are unlikely to be exactly representative of the general population.

### Genotyping and SNP quality control

For the 23andMe cohort, DNA extraction and genotyping were performed on saliva samples by National Genetics Institute (NGI), a CLIA-certified clinical laboratory and subsidiary of Laboratory Corporation of America. Samples were genotyped on the Illumina HumanHap550+ BeadChip platform, which included SNPs from the standard HumanHap550 panel augmented with a custom set of approximately 25,000 SNPs selected by 23andMe. Every sample that failed to reach 98.5% call rate was re-analyzed. Individuals whose analyses failed repeatedly were re-contacted by 23andMe customer service to provide additional samples, as is done for all 23andMe customers. Two slightly different versions of the genotyping platform were used in this study. See [53] for further details on the genotyping and sample quality controls.

The NINDS dataset consisted of 519 samples genotyped on a combination of an Illumina HumanHap 250 K and Illumina HumanHap 300 K chip, and 1,183 samples genotyped on an Illumina HumanHap 550 K chip. As the proportion of cases genotyped on each platform in the NINDS dataset differed between the two platforms, any marker with differing frequencies across the two platforms (due to problems with clustering or other genotyping error) would show up as associated with PD status in the NINDS dataset. To account for potential stratification arising from this, we defined a binary covariate to indicate genotyping platform, which we used for covariate adjustment during analyses involving the NINDS dataset.

In all analyses, SNPs with a call rate 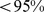 or minor allele frequency 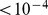 were excluded from analysis. Additionally, SNPs with Hardy-Weinberg 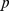-values 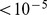 or 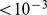 were excluded from the 23andMe and NINDS datasets, respectively [56]. For analyses involving external validation of risk prediction models trained on the 23andMe dataset against the NINDS dataset, only SNPs common to both datasets were used in both model development and testing. Altogether, 522,782 SNPs were retained for the 23andMe dataset with an average call rate of 99.8%, 514,362 SNPs were retained for the NINDS dataset with an average call rate of 99.8%, and 492,136 SNPS were common to both datasets.

### Association analysis

For the association analysis, all 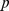-values were calculated using a likelihood ratio test for the logistic regression model, adjusting for sex, age, and the first five principal components (chosen based on an examination of the eigenspectrum of our data):
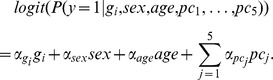
Here, the phenotypic status of each individual was coded as 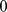 for unaffected individuals and 1 for affected individuals. Genotypes 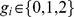 were coded to indicate the number of minor alleles present for tested SNP (corresponding to a log-additive model of association), and 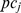 was the projection of the individual onto the 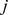
th principal component of the genotype data matrix. Reported odds ratios for each SNP relative to the minor allele were defined as 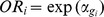, and the alleles used throughout refer to the plus strand of NCBI build 36.3 of the human genome.

Principal components were computed using multi-dimensional scaling over the allele-sharing distance matrix as in [53]. The SNPs used for the replication analysis (Table 3) were taken from http://www.pdgene.org/ [27] and http://www.genome.gov/gwastudies/ [57] on November 18, 2010. We added the SNPs reported as genome-wide significant from [22] to this list. For the power calculations, we used the model from [58].

### Heritability estimation

To estimate heritability of liability, we used the GCTA package (v0.90.3) [29] for genome-wide complex trait analysis. Previously, this approach was used to estimate the proportion of the heritability in height that could be explained by common variation on a genomic panel [69]. Here, we used a recent adaptation of this method to case-control studies [30] to estimate the heritability of PD in both the NINDS and 23andMe cohorts. We analyzed the NINDS cohort data by using GCTA to remove individuals with genetic relationship greater than 0.025, and estimating heritability of liability using 20 principal components as covariates and assuming a disease prevalence of 0.01. For the 23andMe cohort, we adopted the same procedure but pre-filtered the data by stratifying the dataset on sex, and matching on age and five principal components in order to obtain a reduced size dataset with one case per four controls.

For Table 4, we converted relative recurrence risk ratios 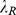 to heritability of liability estimates 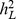 using a modification of the analysis described in [31]. Formulas for estimating the maximum AUC achievable for a given heritability, and for computing the proportion of variance in liability explained were also based on [31]. Details are provided in Text S1.

We note that in Table 4, the heritability estimates shown were based on the authors' criteria for “broad-definition PD” as no heritability estimates could be provided for the strict PD definition due to the lack of concordant monozygotic twins present in the dataset. For [10], 95% confidence intervals were obtained through a reanalysis of the original data using 100,000 bootstrap samples for the counts of doubly-ascertained concordant, singly-ascertained concordant, and discordant monozygotic and dizygotic twin pairs. Heritability of liability was estimated as twice the difference in tetrachoric correlations for monozygotic and dizygotic twins, using a specialized numerical integration procedure for multivariate normal densities [70].

### Risk prediction

We performed risk prediction experiments using a sparse logistic regression solver based on the “elastic net” regularization penalty [59]. In this approach, one solves the convex optimization problem,
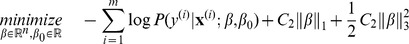
for fixed constants 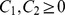, where 
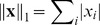
 and 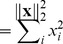, and where 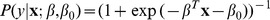. Sparse regression methods, which tend to estimate solution vectors 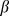 with very few non-zero components, have enjoyed increased popularity in recent years due to their effectiveness in picking out relevant features from extremely high-dimensional data. In the context of genetic association analysis, sparse regression methods can be used to identify the set of SNPs that are most relevant to prediction of a given phenotype. The “elastic net” approach we used is a particular sparse regression variant based on combining 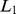/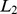 regularization penalties that has the advantage of grouping together correlated features while maintaining sparsity. We note that elastic net regularization has also previously been applied in the context of GWAS and SNP-based risk prediction in a number of recent papers [32], [60]–[62].

For all experiments, we used a default value of 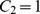 (to ensure uniqueness of the solution to the optimization problem) and evaluated the performance of the risk prediction algorithm in two different ways. We varied the 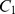 hyperparameter to obtain different bounds on the expected number of false positive associations (i.e., genotype features with non-zero coefficients corresponding to SNPs that are not truly associated with the phenotype), using an adaptation of the analysis from [63].

### AUC analysis

We performed two types of cross-validation experiments: an internal cross-validation analysis using only the 23andMe cohort, and an external cross-validation analysis testing the predictive accuracy of a model trained using the 23andMe cohort on the NINDS cohort. In both cases, all aspects of the analysis following QC were included as part of the cross-validation.

For the internal cross-validation experiment, we generated a matched dataset using a portion of the 23andMe cohort. More specifically, we separated the 23andMe dataset into 20 partitions based on sex and age decile. Next, all partitions were then balanced to contain roughly the same ratio of cases to controls. Finally, five equally-sized cross-validation folds were formed, containing the same amount of representation from each of the partitions. In total 3,380 cases and 21,640 controls were used across the five cross-validation folds. For the external cross-validation experiment, the entire 23andMe cohort was used as a training set, and evaluation was performed on the NINDS dataset.

When estimating AUC using a test set, stratification biases can arise when the apparent discriminative accuracy of the model can be attributed to one or more covariates. This could occur, for example, if the prevalence of the disease were to vary by population, provided that the SNPs included in the risk prediction model were informative of ancestry. To control for confounding from covariate imbalance, we used a stratified variant of the AUC known as the “covariate-adjusted AUC,” defined as the probability that a randomly selected case will have a higher estimated risk of developing PD than a randomly selected *matched* control. More details on our procedure for covariate adjustment are provided in Text S1.

The use of AUC-based statistics for risk prediction is not without controversy. In the setting of a case-control study, the AUC has the advantage of being neither dependent on an arbitrarily set threshold for risk (which would be needed when computing sensitivity or specificity) or the relative proportion of cases and controls in the study. Some authors have contended that the AUC is not a clinically relevant measurement of performance and may be insensitive to changes that would otherwise be considered important in a diagnostic setting [64]–[67], while others have argued that changes in AUC are nonetheless meaningful in assessing discriminative performance [68]. Here, we have chosen to rely on AUC not as a summary of the clinical performance of a classifier, but rather as a mechanism for studying the genetic etiology of a disease, and for estimating the proportion of genetic variance captured by the SNPs used in our models. Were one specifically interested in developing a clinically useful classifier, other measures of accuracy may be more appropriate.

Since the goal of our experiments was to measure the predictive capacity that could be attributed to SNPs in our model, rather than covariates such as sex, age, or ancestry, we intentionally excluded covariates when fitting our predictive models. We note that because of our use of covariate-adjusted AUCs, the decision to exclude covariates had little impact on our results since changes in predictive performance arising from the inclusion of covariates would have been “factored out” by the stratification procedure anyway. For example, the covariate-adjusted external validation accuracies of the smallest and largest models in Table 5 were 0.550 and 0.605, respectively; the analogous risk prediction model including covariates would have achieved accuracies of 0.557 and 0.603.

## Supporting Information

Figure S1Manhattan plot 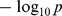-values by chromosome for the 23andMe dataset. Genome-wide significant SNPs are shown in red.(TIFF)

Figure S2Quantile-quantile plot Observed 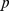-values versus theoretical 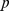-values under the null. The genomic control inflation factor for the study was 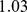 and is shown by the red line.(TIFF)

Figure S3Plot of 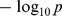-values around *RIT2*/*SYT4*. Colors depict the squared correlation (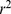) of each SNP with rs4130047. For details, see Figure 1.(TIFF)

Figure S4Plot of 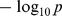-values around rs2823357 and *USP25*. Colors depict the squared correlation (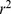) of each SNP with rs2823357. For details, see Figure 1.(TIFF)

Table S1 Genotype by phenotype tables for SNPs in Table 2.
(PDF)

Table S2Details for all SNPs with 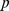-values under 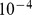. See Table 2 for details.(XLS)

Table S3Internal and external cross-validation AUC difference test for sparse logistic regression models. Rows and columns of each table correspond to models being compared, and are labeled using the theoretical upper bound on 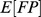 of the model for that particular row or column. Elements of the tables are one-sided 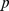-value tests for the alternative hypothesis that the row model has a higher AUC than the column model. One-sided comparisons significant at the 0.05 level are indicated in bold.(PDF)

Table S4External cross-validation AUC difference test using bias-corrected models. Each row of the table represents a comparison of a “test” risk prediction model based on the significance threshold indicated in the first column against a “reference” model containing only SNPs found in genome-wide significant regions. In all cases, reported AUCs have been adjusted for covariates (see Materials and Methods), and all models were bias-corrected by omitting the sparsity-inducing prior during model fitting. The second and third columns show the predicted AUC for each model based on the estimated SNP effect sizes and test distribution genotype frequencies. The fourth and fifth column show the covariate-adjusted AUCs actually observed on the test data. The poor agreement between predicted and observed test AUC for the largest two models is evidence of severe overfitting in these cases. The last column gives one-sided 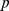-values for an AUC difference test under the alternative hypothesis that the test model has a higher AUC than the reference model; one-sided comparisons nominally significant at the 0.05 level are indicated in bold.(PDF)

Table S5Bias-corrected 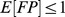 model. This model, which achieves a covariate-adjusted AUC of 0.608 on the NINDS data, was obtained by training on the 23andMe cohort, using the subset of SNPs that were shared with the NINDS cohort. 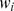 refers to the weight for each SNP (i.e., the log odds ratio per copy of the alphabetically lesser allele), and 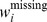 is the weight used in the algorithm in the case of missing data for that SNP.(PDF)

Table S6Bias-corrected 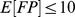 model. This model, which achieves a covariate-adjusted AUC of 0.614 on the NINDS data, was obtained by training on the 23andMe cohort, using the subset of SNPs that were shared with the NINDS cohort. 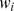 refers to the weight for each SNP (i.e., the log odds ratio per copy of the alphabetically lesser allele), and 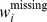 is the weight used in the algorithm in the case of missing data for that SNP.(PDF)

Table S7Test for heterogeneity. The low confidence group consisted of participants who did not answer a questionnaire, whereas the high confidence group consisted of participants who did. The second and third columns show the estimated log odds-ratio for each SNP from Table 2 using only low and high confidence data, respectively. The fourth column shows the combined log odds-ratio when using data from both groups together, and the final column gives a 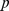-value for heterogeneity. Note that while three of the 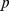-values are nominally significant, only that for rs12185268 survives a correction for multiple testing (correcting for the 11 SNPs tested).(PDF)

Table S8 Exclusionary conditions. People reporting any of the above diagnoses were excluded from the analysis.
(PDF)

Text S1 (PDF)
