# Supplementary figures and images for: Web-Based Genome-Wide Association Study Identifies Two Novel Loci and a Substantial Genetic Component for Parkinson's Disease
Source: PLoS Genet. 2011 Jun 23;7(6):e1002141. doi: 10.1371/journal.pgen.1002141 (PMC3121750; doi:10.1371/journal.pgen.1002141)

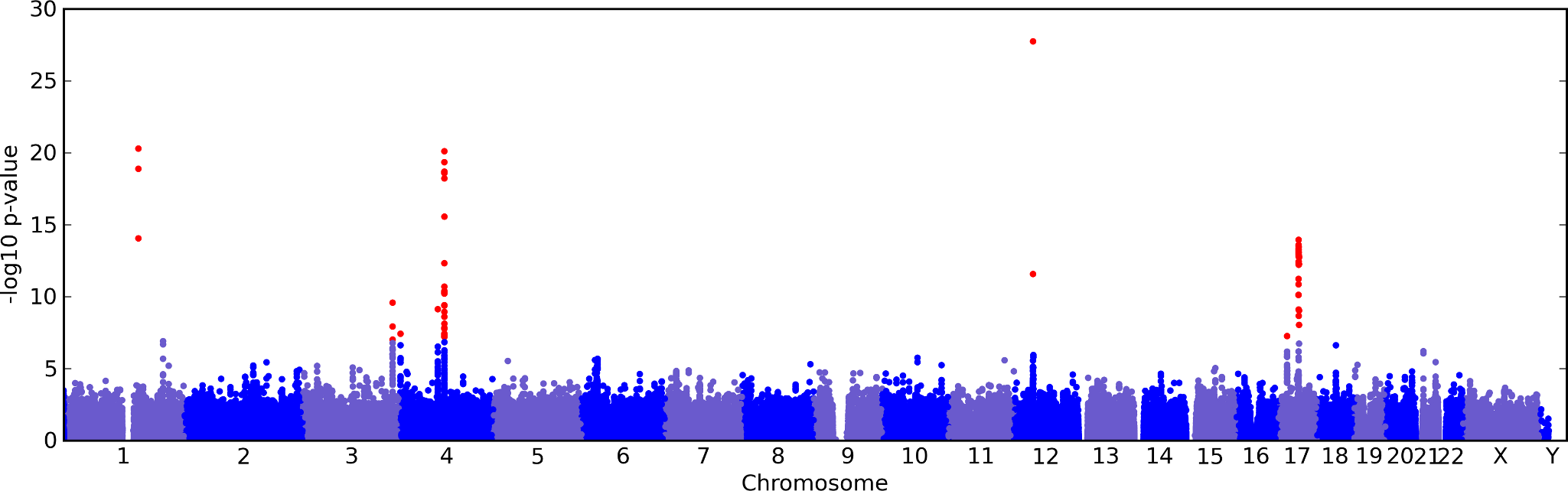

Supplement: Figure S1 — Manhattan plot -values by chromosome for the 23andMe dataset. Genome-wide significant SNPs are shown in red. (TIFF) [file pgen.1002141.s001.tiff]

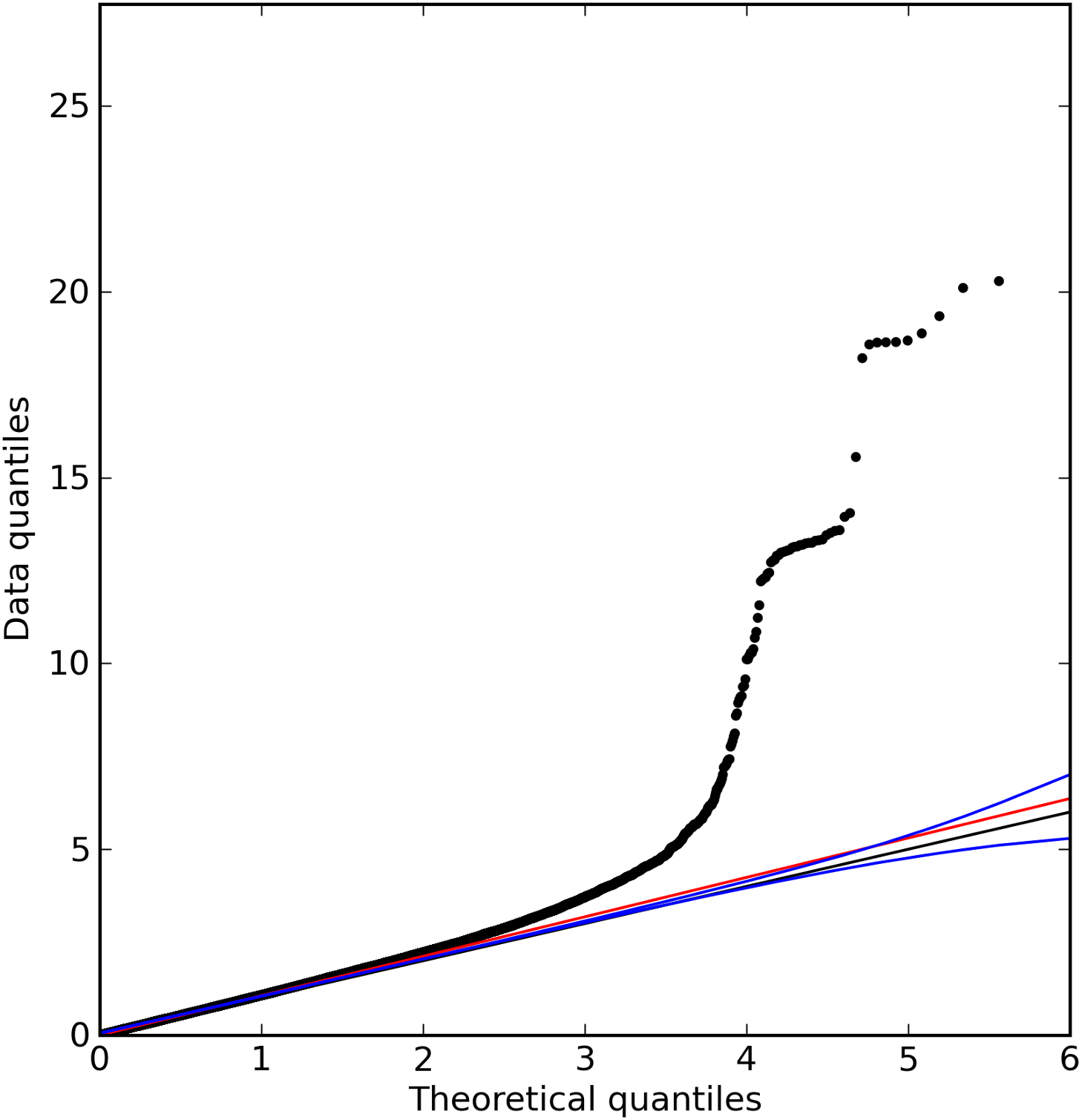

Supplement: Figure S2 — Quantile-quantile plot Observed -values versus theoretical -values under the null. The genomic control inflation factor for the study was and is shown by the red line. (TIFF) [file pgen.1002141.s002.tiff]

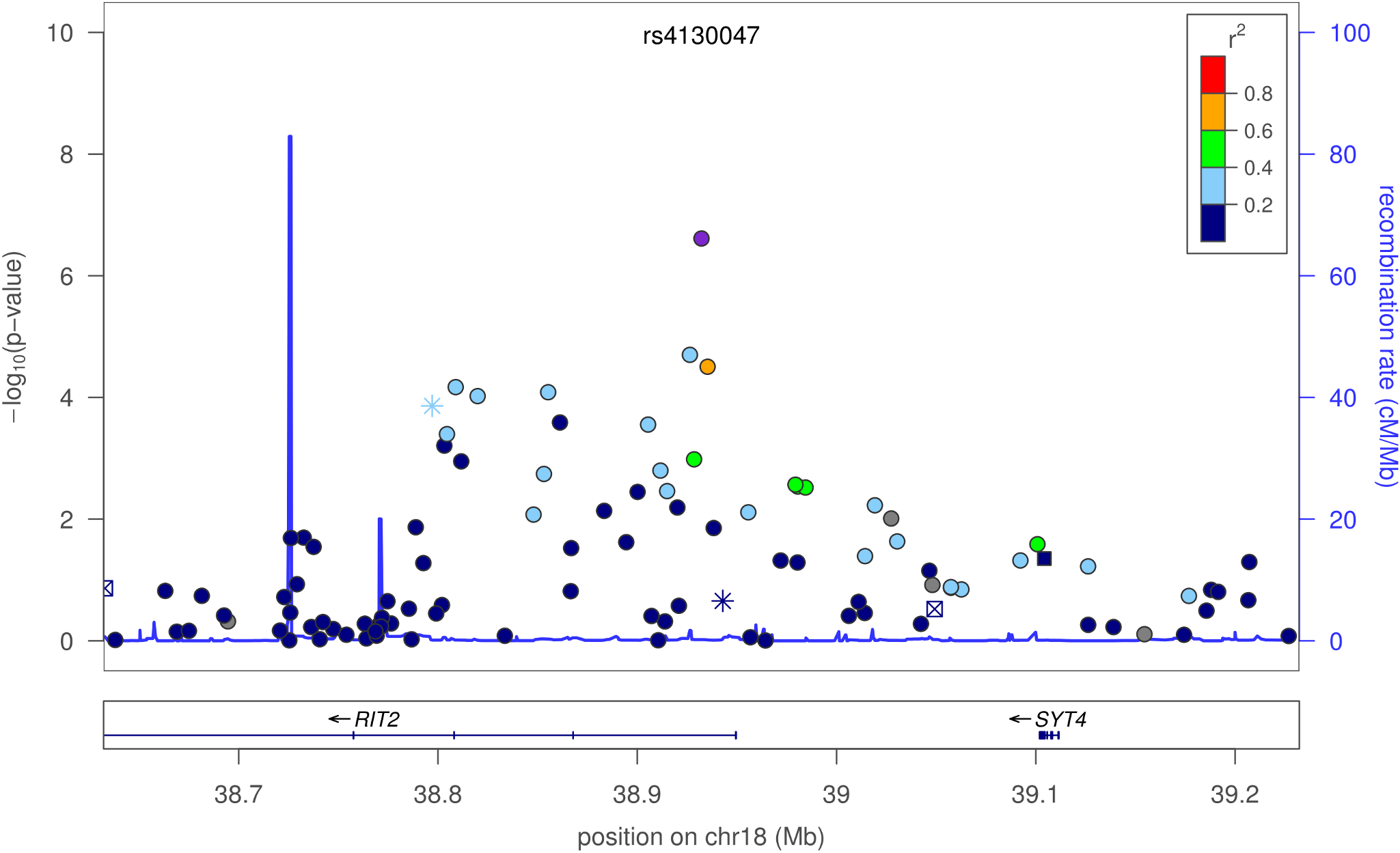

Supplement: Figure S3 — Plot of -values around RIT2/SYT4. Colors depict the squared correlation () of each SNP with rs4130047. For details, see Figure 1. (TIFF) [file pgen.1002141.s003.tiff]

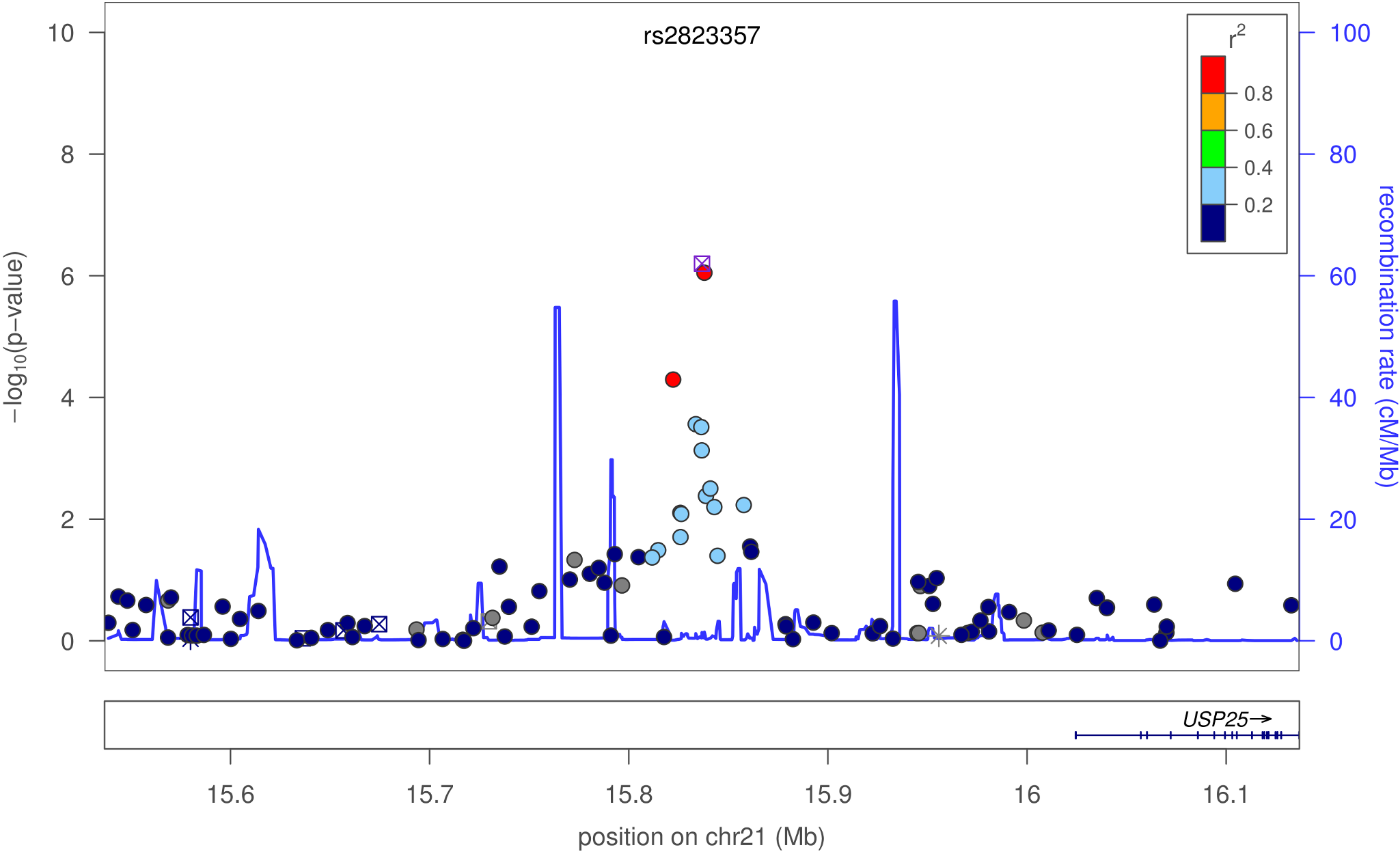

Supplement: Figure S4 — Plot of -values around rs2823357 and USP25. Colors depict the squared correlation () of each SNP with rs2823357. For details, see Figure 1. (TIFF) [file pgen.1002141.s004.tiff]
