# Supplementary material for: Web-Based Genome-Wide Association Study Identifies Two Novel Loci and a Substantial Genetic Component for Parkinson's Disease
Source: PLoS Genet. 2011 Jun 23;7(6):e1002141. doi: 10.1371/journal.pgen.1002141 (PMC3121750; doi:10.1371/journal.pgen.1002141)
Supplement: Table S1 — Genotype by phenotype tables for SNPs in Table 2. (PDF) [file pgen.1002141.s005.pdf]

Web-based genome-wide association study identifies two novel loci and a substantial genetic component for Parkinson's disease

Do, Tung, Dorfman, Kiefer, Drabant, Francke, Mountain, Goldman, Tanner, Langston, Wojcicki, Eriksson

rs34637584	GG (%)	GA (%)	AA (%)
Control	29570 (89.8)	53 (42.1)	1 (50.0)
Case	3352 (10.2)	73 (57.9)	1 (50.0)

i4000416	TT (%)	TC (%)
Control	28399 (89.5)	206 (66.9)
Case	3319 (10.5)	102 (33.1)

rs356220	CC (%)	CT (%)	TT (%)
Control	11792 (91.5)	13789 (88.9)	4014 (86.7)
Case	1094 (8.5)	1713 (11.1)	618 (13.3)

rs12185268	AA (%)	AG (%)	GG (%)
Control	18074 (88.7)	9786 (91.1)	1411 (92.3)
Case	2309 (11.3)	957 (8.9)	117 (7.7)

rs10513789	TT (%)	TG (%)	GG (%)
Control	18770 (88.9)	9572 (90.7)	1266 (92.7)
Case	2339 (11.1)	986 (9.3)	100 (7.3)

rs6812193	CC (%)	CT (%)	TT (%)
Control	11797 (88.5)	13762 (90.1)	4062 (91.6)
Case	1535 (11.5)	1517 (9.9)	373 (8.4)

rs6599389	GG (%)	GA (%)	AA (%)
Control	25412 (90.0)	4017 (87.8)	152 (83.5)
Case	2836 (10.0)	559 (12.2)	30 (16.5)

rs11868035	GG (%)	GA (%)	AA (%)
Control	14038 (88.7)	12626 (90.4)	2921 (91.3)
Case	1795 (11.3)	1345 (9.6)	280 (8.7)

rs823156	AA (%)	AG (%)	GG (%)
Control	19627 (89.0)	8953 (90.8)	1037 (91.8)
Case	2424 (11.0)	908 (9.2)	93 (8.2)

rs4130047	TT (%)	TC (%)	CC (%)
Control	14077 (90.2)	12696 (89.5)	2850 (87.3)
Case	1525 (9.8)	1485 (10.5)	416 (12.7)

rs2823357	GG (%)	GA (%)	AA (%)
Control	11636 (90.3)	13883 (89.6)	4101 (87.8)
Case	1252 (9.7)	1604 (10.4)	570 (12.2)

Table S1. Genotype by phenotype tables for SNPs in Table 2.
