# Supplementary material for: Web-Based Genome-Wide Association Study Identifies Two Novel Loci and a Substantial Genetic Component for Parkinson's Disease
Source: PLoS Genet. 2011 Jun 23;7(6):e1002141. doi: 10.1371/journal.pgen.1002141 (PMC3121750; doi:10.1371/journal.pgen.1002141)
Supplement: Table S3 — Internal and external cross-validation AUC difference test for sparse logistic regression models. Rows and columns of each table correspond to models being compared, and are labeled using the theoretical upper bound on of the model for that particular row or column. Elements of the tables are one-sided -value tests for the alternative hypothesis that the row model has a higher AUC than the column model. One-sided comparisons significant at the 0.05 level are indicated in bold. (PDF) [file pgen.1002141.s007.pdf]

Web-based genome-wide association study identifies two novel loci and a substantial genetic component for Parkinson's disease

Do, Tung, Dorfman, Kiefer, Drabant, Francke, Mountain, Goldman, Tanner, Langston, Wojcicki, Eriksson

Internal

1	**0.024**			
10	**0.000**	**0.000**		
100	**0.000**	**0.000**	**0.026**	
1000	**0.001**	**0.004**	0.075	0.308
	0.05	1	10	100

External

1	**0.025**			
10	**0.009**	0.120		
100	**0.022**	0.179	0.452	
1000	**0.010**	0.081	0.205	0.182
	0.05	1	10	100

Table S3. Internal and external cross-validation AUC difference test for sparse logistic regression models. Rows and columns of each table correspond to models being compared, and are labeled using the theoretical upper bound on $E[FP]$ of the model for that particular row or column. Elements of the tables are one-sided p-value tests for the alternative hypothesis that the row model has a higher AUC than the column model. One-sided comparisons significant at the 0.05 level are indicated in bold.
