# Supplementary material for: Web-Based Genome-Wide Association Study Identifies Two Novel Loci and a Substantial Genetic Component for Parkinson's Disease
Source: PLoS Genet. 2011 Jun 23;7(6):e1002141. doi: 10.1371/journal.pgen.1002141 (PMC3121750; doi:10.1371/journal.pgen.1002141)
Supplement: Table S4 — External cross-validation AUC difference test using bias-corrected models. Each row of the table represents a comparison of a “test” risk prediction model based on the significance threshold indicated in the first column against a “reference” model containing only SNPs found in genome-wide significant regions. In all cases, reported AUCs have been adjusted for covariates (see Materials and Methods), and all models were bias-corrected by omitting the sparsity-inducing prior during model fitting. The second and third columns show the predicted AUC for each model based on the estimated SNP effect sizes and test distribution genotype frequencies. The fourth and fifth column show the covariate-adjusted AUCs actually observed on the test data. The poor agreement between predicted and observed test AUC for the largest two models is evidence of severe overfitting in these cases. The last column gives one-sided -values for an AUC difference test under the alternative hypothesis that the test model has a higher AUC than the reference model; one-sided comparisons nominally significant at the 0.05 level are indicated in bold. (PDF) [file pgen.1002141.s008.pdf]

Web-based genome-wide association study identifies two novel loci and a substantial genetic component for Parkinson's disease

Do, Tung, Dorfman, Kiefer, Drabant, Francke, Mountain, Goldman, Tanner, Langston, Wojcicki, Eriksson

Significance Threshold	$\text{AUC}^{\text{pred}}_{\text{test}}$	$\text{AUC}^{\text{pred}}_{\text{ref}}$	$\text{AUC}^{\text{obs}}_{\text{test}}$	$\text{AUC}^{\text{obs}}_{\text{ref}}$	p
$E[FP] \leq 1$	0.589	0.569	0.608 ± 0.046	0.570 ± 0.051	**0.0389**
$E[FP] \leq 10$	0.627	0.570	0.614 ± 0.043	0.567 ± 0.051	**0.0469**
$E[FP] \leq 100$	0.697	0.572	0.553 ± 0.050	0.559 ± 0.051	0.589
$E[FP] \leq 1000$	0.796	0.573	0.560 ± 0.048	0.571 ± 0.051	0.631

Table S4. External cross-validation AUC difference test using bias-corrected models. Each row of the table represents a comparison of a "test" risk prediction model based on the significance threshold indicated in the first column against a "reference" model containing only SNPs found in genome-wide significant regions. In all cases, reported AUCs have been adjusted for covariates (see Materials and Methods), and all models were bias-corrected by omitting the sparsity-inducing prior during model fitting. The second and third columns show the predicted AUC for each model based on the estimated SNP effect sizes and test distribution genotype frequencies. The fourth and fifth column show the covariate-adjusted AUCs actually observed on the test data. The poor agreement between predicted and observed test AUC for the largest two models is evidence of severe overfitting in these cases. The last column gives one-sided p-values for an AUC difference test under the alternative hypothesis that the test model has a higher AUC than the reference model; one-sided comparisons nominally significant at the 0.05 level are indicated in bold.
