# Supplementary material for: Web-Based Genome-Wide Association Study Identifies Two Novel Loci and a Substantial Genetic Component for Parkinson's Disease
Source: PLoS Genet. 2011 Jun 23;7(6):e1002141. doi: 10.1371/journal.pgen.1002141 (PMC3121750; doi:10.1371/journal.pgen.1002141)
Supplement: Table S5 — Bias-corrected model. This model, which achieves a covariate-adjusted AUC of 0.608 on the NINDS data, was obtained by training on the 23andMe cohort, using the subset of SNPs that were shared with the NINDS cohort. refers to the weight for each SNP (i.e., the log odds ratio per copy of the alphabetically lesser allele), and is the weight used in the algorithm in the case of missing data for that SNP. (PDF) [file pgen.1002141.s009.pdf]

Web-based genome-wide association study identifies two novel loci and a substantial genetic component for Parkinson's disease

Do, Tung, Dorfman, Kiefer, Drabant, Francke, Mountain, Goldman, Tanner, Langston, Wojcicki, Eriksson

rsid	chrom	bp	w_i	$w_i^{missing}$
bias	-	-	-1.93614	-
rs823156	1	204031263	0.17978	0.29361
rs2117511	2	136179131	-0.19903	-0.04755
rs10513789	3	184242767	-0.20692	-0.08325
rs6599389	4	929113	0.22544	0.03374
rs11248060	4	954359	-0.18680	-0.32637
rs11724804	4	955779	-0.07805	-0.06768
rs4395500	4	13433745	-0.15007	-0.23841
rs6812193	4	77418010	0.17122	0.21732
rs11931074	4	90858538	-0.14634	-0.26935
rs356220	4	90860363	-0.11414	-0.14267
rs2736990	4	90897564	-0.13371	-0.14259
rs9366778	6	31377152	-0.12434	-0.10019
rs10886515	10	121333579	-0.15044	-0.08701
rs6589497	11	115128203	0.18249	0.04090
rs10878246	12	38918366	0.15039	0.05717
rs11868035	17	17655826	-0.15247	-0.09411
rs708382	17	39797870	0.12370	0.09699
rs12185268	17	41279463	0.17776	0.28040
rs12373139	17	41279910	-0.06829	-0.02925
rs11878694	19	6947509	0.20529	0.36115
rs1276377	20	55031299	-0.08092	-0.10845
rs331617	20	55073255	0.09799	0.05514

Table S5. Bias-corrected $E[FP] \leq 1$ model. This model, which achieves a covariate-adjusted AUC of 0.608 on the NINDS data, was obtained by training on the 23andMe cohort, using the subset of SNPs that were shared with the NINDS cohort. w_i refers to the weight for each SNP (i.e., the log odds ratio per copy of the alphabetically lesser allele), and w_i^{missing} is the weight used in the algorithm in the case of missing data for that SNP.
