# Supplementary material for: Web-Based Genome-Wide Association Study Identifies Two Novel Loci and a Substantial Genetic Component for Parkinson's Disease
Source: PLoS Genet. 2011 Jun 23;7(6):e1002141. doi: 10.1371/journal.pgen.1002141 (PMC3121750; doi:10.1371/journal.pgen.1002141)
Supplement: Table S6 — Bias-corrected model. This model, which achieves a covariate-adjusted AUC of 0.614 on the NINDS data, was obtained by training on the 23andMe cohort, using the subset of SNPs that were shared with the NINDS cohort. refers to the weight for each SNP (i.e., the log odds ratio per copy of the alphabetically lesser allele), and is the weight used in the algorithm in the case of missing data for that SNP. (PDF) [file pgen.1002141.s010.pdf]

Web-based genome-wide association study identifies two novel loci and a substantial genetic component for Parkinson's disease

Do, Tung, Dorfman, Kiefer, Drabant, Francke, Mountain, Goldman, Tanner, Langston, Wojcicki, Eriksson

rsid	chrom	bp	w_i	$w_i^{missing}$
bias	-	-	-3.90034	-
rs6576808	1	86411336	0.19929	0.35684
rs823156	1	204031263	0.17777	0.29033
rs6680316	1	220023249	-0.20185	-0.37328
rs2117511	2	136179131	-0.19897	-0.04754
rs1234413	2	148560839	0.11041	0.12413
rs9917256	2	168851281	0.17036	0.04290
rs1020357	3	55318983	0.20863	0.03001
rs4076437	3	130518659	0.16674	0.03897
rs10513789	3	184242767	-0.21099	-0.08489
rs6599389	4	929113	0.22623	0.03386
rs11248060	4	954359	-0.17194	-0.30041
rs11724804	4	955779	-0.07200	-0.06244
rs4395500	4	13433745	-0.14916	-0.23697
rs4698412	4	15346446	0.11337	0.12498
rs10517056	4	24494382	-0.12298	-0.07220
rs7655536	4	77395792	0.09867	0.03925
rs6812193	4	77418010	0.14010	0.17782
rs356229	4	90825620	0.09754	0.07168
rs11931074	4	90858538	-0.16114	-0.29658
rs356220	4	90860363	-0.05023	-0.06279
rs2736990	4	90897564	-0.11748	-0.12527
rs4397141	5	29647646	0.22811	0.41922
rs3869109	6	31292175	0.04131	0.03464
rs9366778	6	31377152	-0.07018	-0.05655
rs659445	6	31972283	-0.08960	-0.12489
rs2072369	7	23112714	0.11663	0.14646
rs6954831	7	36428666	0.21567	0.02441
rs11971935	7	127209775	0.10319	0.08494
rs732468	8	4285284	0.11018	0.06246
rs11775723	8	4997737	-0.14006	-0.22884
rs1486803	9	11706862	-0.12592	-0.09178
rs3740304	10	313283	0.13577	0.19992
rs17632029	10	14008131	0.17250	0.29483
rs3844118	10	78840719	-0.20969	-0.03939
rs10886515	10	121333579	-0.14518	-0.08396
rs12360601	11	20008788	0.11898	0.17624
rs6589497	11	115128203	0.19227	0.04309
rs1503415	11	120991762	0.11567	0.15955
rs10878246	12	38918366	0.09281	0.03528
rs11564252	12	39100000	0.10261	0.03664
rs278901	12	39688545	0.26682	0.02419
rs764902	12	128874436	-0.12846	-0.09505
rs10151526	14	33487738	0.28679	0.01368
rs1885747	14	92117208	-0.14963	-0.24032
rs2414739	15	59781426	0.13622	0.20016
rs3812945	15	73076775	0.12188	0.10963
rs11868035	17	17655826	-0.15142	-0.09347
rs708382	17	39797870	0.11873	0.09308
rs12185268	17	41279463	0.17404	0.27453
rs12373139	17	41279910	0.06091	0.02609
rs7225002	17	41544850	0.06612	0.08045
rs415430	17	42214305	-0.10118	-0.04108
rs12451779	17	55875936	0.23160	0.01986
rs4130047	18	38932233	0.12908	0.08090
rs3943675	18	66201794	-0.11085	-0.10047
rs11878694	19	6947509	0.20332	0.35769
rs1276377	20	55031299	-0.06321	-0.08472
rs331617	20	55073255	0.10635	0.05984
rs2823357	21	15836776	0.10946	0.08224
rs138054	22	42550905	0.12893	0.05339

Table S6. Bias-corrected $E[FP] \leq 10$ model. This model, which achieves a covariate-adjusted AUC of 0.614 on the NINDS data, was obtained by training on the 23andMe cohort, using the subset of SNPs that were shared with the NINDS cohort. w_i refers to the weight for each SNP (i.e., the log odds ratio per copy of the alphabetically lesser allele), and w_i^{missing} is the weight used in the algorithm in the case of missing data for that SNP.
