# Supplementary material for: Web-Based Genome-Wide Association Study Identifies Two Novel Loci and a Substantial Genetic Component for Parkinson's Disease
Source: PLoS Genet. 2011 Jun 23;7(6):e1002141. doi: 10.1371/journal.pgen.1002141 (PMC3121750; doi:10.1371/journal.pgen.1002141)
Supplement: Table S7 — Test for heterogeneity. The low confidence group consisted of participants who did not answer a questionnaire, whereas the high confidence group consisted of participants who did. The second and third columns show the estimated log odds-ratio for each SNP from Table 2 using only low and high confidence data, respectively. The fourth column shows the combined log odds-ratio when using data from both groups together, and the final column gives a -value for heterogeneity. Note that while three of the -values are nominally significant, only that for rs12185268 survives a correction for multiple testing (correcting for the 11 SNPs tested). (PDF) [file pgen.1002141.s011.pdf]

Web-based genome-wide association study identifies two novel loci and a substantial genetic component for Parkinson's disease

Do, Tung, Dorfman, Kiefer, Drabant, Francke, Mountain, Goldman, Tanner, Langston, Wojcicki, Eriksson

SNP	Low	High	Combined	p-value
rs34637584	-1.153 (-1.79 - -0.52)	-2.100 (-2.50 - -1.70)	-2.263 (-2.66 - -1.86)	0.859
i4000416	-1.441 (-1.89 - -0.99)	-1.162 (-1.46 - -0.87)	-1.398 (-1.67 - -1.12)	0.875
rs356220	0.220 (0.09 - 0.35)	0.244 (0.19 - 0.30)	0.251 (0.20 - 0.31)	0.652
rs12185268	-0.043 (-0.20 - 0.12)	-0.297 (-0.37 - -0.22)	-0.263 (-0.33 - -0.19)	0.002
rs10513789	0.284 (0.11 - 0.46)	0.198 (0.12 - 0.27)	0.220 (0.15 - 0.29)	0.090
rs6812193	-0.037 (-0.17 - 0.10)	-0.195 (-0.26 - -0.13)	-0.176 (-0.23 - -0.12)	0.025
rs6599389	-0.286 (-0.50 - -0.07)	-0.252 (-0.35 - -0.15)	-0.271 (-0.36 - -0.18)	0.193
rs11868035	0.062 (-0.08 - 0.20)	0.175 (0.11 - 0.24)	0.162 (0.10 - 0.22)	0.013
rs823156	-0.180 (-0.35 - -0.01)	-0.183 (-0.26 - -0.11)	-0.190 (-0.26 - -0.12)	0.937
rs4130047	-0.131 (-0.27 - 0.00)	-0.145 (-0.20 - -0.08)	-0.149 (-0.21 - -0.09)	0.257
rs2823357	-0.234 (-0.36 - -0.11)	-0.112 (-0.17 - -0.05)	-0.139 (-0.19 - -0.08)	0.277

Table S7. Test for heterogeneity. The low confidence group consisted of participants who did not answer a questionnaire, whereas the high confidence group consisted of participants who did. The second and third columns show the estimated log odds-ratio for each SNP from Table 2using only low and high confidence data, respectively. The fourth column shows the combined log odds-ratio when using data from both groups together, and the final column gives a p-value for heterogeneity. Note that while three of the p-values are nominally significant, only that for rs12185268 survives a correction for multiple testing (correcting for the 11 SNPs tested).
