# Supplementary material for: Web-Based Genome-Wide Association Study Identifies Two Novel Loci and a Substantial Genetic Component for Parkinson's Disease
Source: PLoS Genet. 2011 Jun 23;7(6):e1002141. doi: 10.1371/journal.pgen.1002141 (PMC3121750; doi:10.1371/journal.pgen.1002141)
Supplement: Table S8 — Exclusionary conditions. People reporting any of the above diagnoses were excluded from the analysis. (PDF) [file pgen.1002141.s012.pdf]

Web-based genome-wide association study identifies two novel loci and a substantial genetic component for Parkinson's disease

Do, Tung, Dorfman, Kiefer, Drabant, Francke, Mountain, Goldman, Tanner, Langston, Wojcicki, Eriksson

Progressive supranuclear palsy
Multiple system atrophy (or Shy-Drager syndrome or striatonigral degeneration)
Spinal cerebellar ataxia (or cerebellar degeneration or olivopontocerebellar atrophy)
Cortical basal ganglionic degeneration
Parkinsonism due to medications (or drug-induced parkinsonism)
Frontotemporal dementia with parkinsonism
Frontotemporal dementia
Picks disease
Dystonic tremor
Rubral tremor
Primary writing tremor
Cerebellar tremor
Multiple sclerosis
Motor neuron disease (or amyotrophic lateral sclerosis or Lou Gehrig's disease)
Spinal muscular atrophy (or progressive muscular atrophy or primary lateral sclerosis)
Epilepsy (or seizure disorder)
Myoclonus
Dystonia
Tourette syndrome (or tic disorder)

Table S8. Exclusionary conditions. People reporting any of the above diagnoses were excluded from the analysis.
