# Supplementary material for: Web-Based Genome-Wide Association Study Identifies Two Novel Loci and a Substantial Genetic Component for Parkinson's Disease
Source: PLoS Genet. 2011 Jun 23;7(6):e1002141. doi: 10.1371/journal.pgen.1002141 (PMC3121750; doi:10.1371/journal.pgen.1002141)
Supplement: Text S1 — (PDF) [file pgen.1002141.s013.pdf]

Web-based genome-wide association study identifies two novel loci and a substantial genetic component for Parkinson's disease

Do, Tung, Dorfman, Kiefer, Drabant, Francke, Mountain, Goldman, Tanner, Langston, Wojcicki, Eriksson

Supplemental Text

Population stratification

For all evaluations of risk prediction models, we controlled for population stratification using a subclassification technique based on full matching. To be conservative, we included any covariate for which we suspected that stratification might be a concern, erring on the side of being more, rather than less, inclusive. For example, we used sex as a covariate to ensure that differences in sex distribution between training and testing sets would not affect the evaluation accuracy, given that we did not explicitly exclude sex-related markers from our predictive models. Similarly, we corrected for age to avoid potential confounding due to the age-dependent penetrance of PD. Stratifying on covariates reduces the bias of our accuracy estimates at the expense of somewhat increased variance. In the remainder of this section, we describe the details of our stratification approach.

Specifically, we partitioned the 23andMe cohort based on sex and cross-validation fold, and partitioned the NINDS cohort based on sex and genotyping platform. Next, we applied an "optimal" subclassification technique in which each partition was further divided into strata, according to the following requirements: each individual in the partition should be assigned to exactly one stratum; each stratum should contain at least one case and one control; and the sum of the distances between cases and controls over all strata should be minimized. For the distance function, we used the Mahalanobis distance metric, $d(\mathbf{u}, \mathbf{v}) = \sqrt{(\mathbf{u}-\mathbf{v})^\intercal \mathbf{\Sigma}^{-1}(\mathbf{u}-\mathbf{v})}$, where $\mathbf{u}$ and $\mathbf{v}$ are vectors containing the age of the individual at collection and the first five principal component projections for a pair of individuals, and where $\mathbf{\Sigma}$ is the pooled covariance matrix estimated from the ages and principal components of all individuals in a given partition. To ensure that individuals within a given partition did not differ too substantially in terms of age or ancestry, we imposed a maximum limit of 0.5 on the Mahalanobis distance, such that individuals with no sufficiently close matches were omitted from the evaluation.

Using the distance function above, there always exists an optimal subclassification in which each stratum contains either exactly one case or exactly one control (known as a "full matching"), and such a subclassification may be found by reducing the distance optimization task to an instance of a min-cost flow problem, for which standard solution techniques exist [1]. For this work, we used a standard min-cost flow algorithm based on successive shortest paths [2], modified to incorporate the labeling heuristic described in [3].

Given the above division of an evaluation dataset into strata, we then performed a stratified receiver operating characteristic (ROC) curve analysis by computing a pooled covariate-adjusted area under curve (AUC) statistic:

$$\hat{A} = \frac{1}{\sum_k |X_k||Y_k|} \sum_k |X_k||Y_k| \hat{A}(X_k, Y_k),$$

where X_k is the set of cases in the kth stratum, Y_k is the set of controls in the kth stratum, and $\hat{A}(X_k, Y_k)$ is the estimated AUC for the kth stratum. Details of the AUC estimation procedure and the precise formulation we used are provided in the next section.

In this formulation, cases in a given subclassification stratum are only compared against controls in the same stratum. In situations where a limited number of strata are used, subclassification can lead to biased estimates if there exists residual covariate imbalance within strata [4]; however, full matching

substantially ameliorates these effects since the small strata ensure that the cases and controls are as similar as possible with respect to observed covariates. For significance testing, we analytically computed unbiased estimates of the variance of the AUC statistic for a given risk prediction model, and the variance of the difference of AUC statistics for two risk prediction models over the same set of strata.

The covariate-adjustment framework we describe here is largely identical in motivation with the covariate-adjusted ROC analysis described in [5]; we note that the semiparametric estimator described in the latter may provide a suitable drop-in replacement for the subclassification-based approach we have described here that avoids the need for matching.

AUC estimation

The AUC is the probability that a randomly chosen case will have greater risk than a randomly chosen matched control (awarding half-credit in the event of ties). Mathematically, we can define the AUC as $A = \frac{1}{2}(R+1)$ where $R = P(x > y) - P(y > x)$. Here, we consider the following two problems:

1. Estimating the AUC for a genetic risk prediction model, given a stratified dataset $\mathcal{D} = \{(X_k, Y_k)\}_{k=1}^{K}$, where X_k and Y_k denote the set of indices of cases and controls in the kth stratum; and
2. Testing the significance of a difference in the AUCs for two different risk prediction models evaluated on the same stratified dataset.

We provide analytical formulas for obtaining unbiased estimates of the variance for the area under a covariate-adjusted ROC curve, and the variance of the difference in areas between two covariate-adjusted ROC curves.

Covariate-adjusted AUC estimator

Let x_i and y_j denote the predicted risks for the ith case and jth control, respectively. Define $\hat{A}(x, y) = \frac{1}{2}(\hat{R}(x, y) + 1)$ where $\hat{R}(x, y) = \mathbf{I}\{x > y\} - \mathbf{I}\{y > x\}$, and consider the estimator

$$\hat{A} = \frac{1}{\sum_k |X_k||Y_k|} \sum_k \sum_{i \in X_k} \sum_{j \in Y_k} \hat{A}(x_i, y_j). \tag{1}$$

It is straightforward to see that $E[\hat{A}] = A$.

Covariance of two covariate-adjusted AUC estimates

Now, let x'_i and y'_j denote a second set of predicted risks for the ith case and jth control, respectively, and suppose that A' is the AUC measured using these alternate risk estimates (note that we do not exclude the possibility that $(x'_i, y'_i) = (x_i, y_i)$ for all i). We wish to compute

$$\mathrm{Cov}(\hat{A}, \hat{A}') = \frac{1}{4}\,\mathrm{Cov}(\hat{R}, \hat{R}') = \frac{\sum_k \left[\sum_{(i,j)} \sum_{(i',j')} \mathrm{Cov}(\hat{R}(x_i, y_j), \hat{R}(x'_{i'}, y'_{j'}))\right]}{4(\sum_k |X_k||Y_k|)^2}.$$

Note that

$$\begin{aligned}
&\mathrm{Cov}(\hat{R}(x_i,y_j),\hat{R}(x'_{i'},y'_{j'}))\\
&\quad= E[\hat{R}(x_i,y_j),\hat{R}(x'_{i'},y'_{j'})] - E[\hat{R}(x_i,y_j)]E[\hat{R}(x'_{i'},y'_{j'})]\\
&\quad=\begin{cases}
P(x_i>y_j\wedge x'_i>y'_j)+P(x_i<y_j\wedge x'_i<y'_j) & \\
\qquad -P(x_i>y_j\wedge x'_i<y'_j)-P(x_i<y_j\wedge x'_i>y'_j)-R\cdot R' & \text{if } i=i' \text{ and } j=j'\\
P(x_i>y_j\wedge x'_{i'}>y'_j)+P(x_i<y_j\wedge x'_{i'}<y'_j) & \\
\qquad -P(x_i>y_j\wedge x'_{i'}<y'_j)-P(x_i<y_j\wedge x'_{i'}>y'_j)-R\cdot R' & \text{if } i\neq i' \text{ and } j=j'\\
P(x_i>y_j\wedge x'_i>y'_{j'})+P(x_i<y_j\wedge x'_i<y'_{j'}) & \\
\qquad -P(x_i>y_j\wedge x'_i<y'_{j'})-P(x_i<y_j\wedge x'_i>y'_{j'})-R\cdot R' & \text{if } i=i' \text{ and } j\neq j'\\
0 & \text{otherwise}
\end{cases}\\
&\quad=\begin{cases}
B_{XY}-R\cdot R' & \text{if } i=i' \text{ and } j=j'\\
B_{XXY}-R\cdot R' & \text{if } i\neq i' \text{ and } j=j'\\
B_{YYX}-R\cdot R' & \text{if } i=i' \text{ and } j\neq j'\\
0 & \text{otherwise}
\end{cases}
\end{aligned}$$

where

$$\begin{aligned}
B_{XY} &= P(x_i>y_j\wedge x'_i>y'_j)+P(x_i<y_j\wedge x'_i<y'_j)-P(x_i>y_j\wedge x'_i<y'_j)-P(x_i<y_j\wedge x'_i>y'_j)\\
B_{XXY} &= P(x_i>y_j\wedge x'_{i'}>y'_j)+P(x_i<y_j\wedge x'_{i'}<y'_j)-P(x_i>y_j\wedge x'_{i'}<y'_j)-P(x_i<y_j\wedge x'_{i'}>y'_j)\\
B_{YYX} &= P(x_i>y_j\wedge x'_i>y'_{j'})+P(x_i<y_j\wedge x'_i<y'_{j'})-P(x_i>y_j\wedge x'_i<y'_{j'})-P(x_i<y_j\wedge x'_i>y'_{j'}).
\end{aligned}$$

It follows that

$$\mathrm{Cov}(\hat{A},\hat{A}') = \frac{\sum_k |X_k||Y_k|\big[B_{XY}+(|X_k|-1)B_{XXY}+(|Y_k|-1)B_{YYX}-4(|X_k|+|Y_k|-1)(A-\frac{1}{2})(A'-\frac{1}{2})\big]}{4(\sum_k |X_k||Y_k|)^2}.$$

Now, define[1]

$$\begin{aligned}
\hat{B}_{XY} &= \frac{\sum_{k,i,j}\mathbf{I}\{x_i>y_j\wedge x'_i>y'_j\}+\mathbf{I}\{x_i<y_j\wedge x'_i<y'_j\}-\mathbf{I}\{x_i>y_j\wedge x'_i<y'_j\}-\mathbf{I}\{x_i<y_j\wedge x'_i>y'_j\}}{\sum_k |X_k||Y_k|}\\
\hat{B}_{XXY} &= \frac{\sum_{k,i,j,i'\neq i}\mathbf{I}\{x_i>y_j\wedge x'_{i'}>y'_j\}+\mathbf{I}\{x_i<y_j\wedge x'_{i'}<y'_j\}-\mathbf{I}\{x_i>y_j\wedge x'_{i'}<y'_j\}-\mathbf{I}\{x_i<y_j\wedge x'_{i'}>y'_j\}}{\sum_k |X_k||Y_k||X_k-1|}\\
\hat{B}_{YYX} &= \frac{\sum_{k,i,j,j'\neq j}\mathbf{I}\{x_i>y_j\wedge x'_i>y'_{j'}\}+\mathbf{I}\{x_i<y_j\wedge x'_i<y'_{j'}\}-\mathbf{I}\{x_i>y_j\wedge x'_i<y'_{j'}\}-\mathbf{I}\{x_i<y_j\wedge x'_i>y'_{j'}\}}{\sum_k |X_k||Y_k||Y_k-1|},
\end{aligned}$$

[1] Naively, the computation of $\hat{B}_{XXY}$ and $\hat{B}_{YYX}$ would take $O(\sum_k |X_k|^2|Y_k|)$ and $O(\sum_k |X_k||Y_k|^2)$ time. However, this can be sped up considerably by noting that if we define

$$C=\sum_{k,i,j}(\mathbf{I}\{x_i>y_j\}-\mathbf{I}\{x_i<y_j\})(\mathbf{I}\{x'_i>y'_j\}-\mathbf{I}\{x'_i<y'_j\})$$

then

$$\begin{aligned}
\hat{B}_{XY} &= \frac{C}{\sum_k |X_k||Y_k|}\\
\hat{B}_{XXY} &= \frac{\sum_{k,j}\left(\sum_i(\mathbf{I}\{x_i>y_j\}-\mathbf{I}\{x_i<y_j\})\right)\Big(\sum_{i'}(\mathbf{I}\{x'_{i'}>y'_j\}-\mathbf{I}\{x'_{i'}<y'_j\})\Big)-C}{\sum_k |X_k||Y_k||X_k-1|}\\
\hat{B}_{YYX} &= \frac{\sum_{k,i}\Big(\sum_j(\mathbf{I}\{x_i>y_j\}-\mathbf{I}\{x_i<y_j\})\Big)\Big(\sum_{j'}(\mathbf{I}\{x'_{i'}>y'_j\}-\mathbf{I}\{x'_{i'}<y'_j\})\Big)-C}{\sum_k |X_k||Y_k||Y_k-1|}.
\end{aligned}$$

In this form, the relationship of the proposed estimator with the estimate given in [6] is evident.

and let

$$\hat{C}_{\hat{A},\hat{A}'} = \frac{\sum_k |X_k||Y_k|\Big[\hat{B}_{XY} + (|X_k|-1)\hat{B}_{XXY} + (|Y_k|-1)\hat{B}_{YYX} - 4(|X_k|+|Y_k|-1)(\hat{A}-\frac{1}{2})(\hat{A}'-\frac{1}{2})\Big]}{4(\sum_k |X_k||Y_k|)^2}.$$

Since $\hat{B}_{XY}$, $\hat{B}_{XXY}$, and $\hat{B}_{YYX}$ are unbiased estimators of B_{XY}, B_{XXY} and B_{YYX}, respectively, and since since $E[(\hat{A}-\frac{1}{2})(\hat{A}'-\frac{1}{2})] = (A-\frac{1}{2})(A'-\frac{1}{2}) + \operatorname{Cov}(\hat{A},\hat{A}')$, it follows that

$$\begin{aligned} E[\hat{C}_{\hat{A},\hat{A}'}] &= E\left[\frac{\sum_k |X_k||Y_k|\Big(\hat{B}_{XY} + (|X_k|-1)\hat{B}_{XXY} + (|Y_k|-1)\hat{B}_{YYX} - 4(|X_k|+|Y_k|-1)(\hat{A}-\frac{1}{2})(\hat{A}'-\frac{1}{2})\Big)}{4(\sum_k |X_k||Y_k|)^2}\right] \\ &= \frac{\sum_k |X_k||Y_k|\Big(B_{XY} + (|X_k|-1)B_{XXY} + (|Y_k|-1)B_{YYX} - 4(|X_k|+|Y_k|-1)\Big[(A-\frac{1}{2})(A'-\frac{1}{2}) + \operatorname{Cov}(\hat{A},\hat{A}')\Big]}{4(\sum_k |X_k||Y_k|)^2} \\ &= \operatorname{Cov}(\hat{A},\hat{A}') - \frac{\sum_k |X_k||Y_k|(|X_k|+|Y_k|-1)\operatorname{Cov}(\hat{A},\hat{A}')}{(\sum_k |X_k||Y_k|)^2} \\ &= \operatorname{Cov}(\hat{A},\hat{A}')\left[1 - \frac{\sum_k |X_k||Y_k|(|X_k|+|Y_k|-1)}{(\sum_k |X_k||Y_k|)^2}\right]. \end{aligned}$$

Therefore,

$$\hat{C}_{\hat{A},\hat{A}'}\left[1 - \frac{\sum_k |X_k||Y_k|(|X_k|+|Y_k|-1)}{(\sum_k |X_k||Y_k|)^2}\right]^{-1}$$

is an unbiased estimator of $\operatorname{Cov}(\hat{A},\hat{A}')$.

Applications

The variance of an AUC estimator can be found using the formula derived in the previous section as $\operatorname{Var}(\hat{A}) = \operatorname{Cov}(\hat{A},\hat{A})$. In this case, B_{XY}, B_{XXY}, and B_{YYX} simplify as:

$$\begin{aligned} B_{XY} &= P(x_i \neq y_j) \\ B_{XXY} &= P(y_j > x_i \wedge y_j > x_{i'}) + P(y_j < x_i \wedge y_j < x_{i'}) - P(x_i < y_j < x_{i'}) - P(x_{i'} < y_j < x_i) \\ B_{YYX} &= P(x_i > y_j \wedge x_i > y_{j'}) + P(x_i < y_j \wedge x_i < y_{j'}) - P(y_j < x_i < y_{j'}) - P(y_{j'} < x_i < y_j), \end{aligned}$$

and the method described here reduces to a previously described approach [7]. For evaluating the difference between the areas of two risk prediction models,

$$\operatorname{Var}(\hat{A}-\hat{A}') = \operatorname{Var}(\hat{A}) + \operatorname{Var}(\hat{A}') - 2\operatorname{Cov}(\hat{A},\hat{A}').$$

Each of the variances and covariances can be computed using the formula derived above; this approach can be thought of as an unbiased version of the estimator described in [6].

Given the above calculations, we can compute a two-sided p-value for an AUC difference based on the asymptotic result, $\frac{(\hat{A}-\hat{A}')^2}{\operatorname{Var}(\hat{A}-\hat{A}')} \sim \chi^2_1$, which can then be converted to a one-sided p-value for the alternative hypothesis $\hat{A}-\hat{A}' > 0$ by the transformation

$$p_1 = \begin{cases} \frac{1}{2}p_2 & \text{if } \hat{A}-\hat{A}' \geq 0 \\ 1-\frac{1}{2}p_2 & \text{otherwise.} \end{cases}$$

Predicted AUCs

To compute predicted covariate-adjusted AUCs, we used a heuristic approach in which we pooled the cases and controls within each stratum. The predicted AUC for a given stratum was calculated by taking the area under the ROC curve whose points were determined by the expected numbers of cases and controls with risk less than some varying threshold; here, the expectations were based on the predicted probabilities of developing the disease given by the model being evaluated. The pooled estimator was then taken to be the weighted average of the predicted AUCs for each stratum, where each stratum was weighted in proportion to the product of the expected number of cases and controls in that stratum.

We note that the relationship between the predicted AUCs described here and the observed AUC estimator in (1) is that the former relies on expected numbers of cases and controls, whereas the latter can be thought of the analogous quantity based on observed numbers of cases and controls.

Heritability calculations

To convert a relative recurrence risk ratio λ_R to a heritability estimate h_L^2, we assumed a liability threshold model [8] where the liability of an individual is the sum of two independent random variables corresponding to additive genetic and environmental effects, $P = A + E$ where $A \sim \mathcal{N}(0, h_L^2)$ and $E \sim \mathcal{N}(0, 1 - h_L^2)$. Here, h_L^2 is the heritability of liability, and individuals are considered affected whenever $P > T$ where $T = \Phi^{-1}(1 - K)$, Φ is the cdf of a standard normal distribution, and K is the prevalence of the disease.

Letting $P_R = A_R + E_R$ be the decomposition of the liability for a relative of the individual, we assume that (P, P_R) have a joint bivariate normal distribution with correlation ρ, from which it follows that

$$\rho = \text{Cov}(P, P_R) = \text{Cov}(A, A_R) = r h_L^2 \quad \Rightarrow \quad h_L^2 = \frac{\rho}{r}$$

where r is the coefficient of relationship ($\frac{1}{2}$ in the case of parents or siblings); here, the second equality assumes the absence of dominance effects and interactions (which were not included in the model) and the lack of shared environmental covariance (i.e., $\text{Cov}(E, E_R) = 0$). To determine ρ, we numerically solved the equation, $\lambda_R K = P(P_R > T | P > T)$. We note that this procedure yields essentially identical results to the technique described in [9] for the range of parameters used in this study, but makes fewer assumptions in its derivation.

To compute an estimate of the maximum AUC possible for a given heritability of liability, and to estimate the proportion $h_{L[x]}^2$ of variance in liability explained by the SNPs in a genetic profile x, we used the equations derived in [9]; the exact equations used are shown below for reproducibility:

$$\begin{aligned}
i &= \frac{1}{\sqrt{2\pi}K} \exp\left(-\frac{1}{2}T^2\right) \\
v &= \frac{-1}{\sqrt{2\pi}(1-K)} \exp\left(-\frac{1}{2}T^2\right) \\
\text{AUC}_{\max} &= \Phi\left(\frac{(i-v)h_L^2}{\sqrt{h_L^2(2 - h_L^2(i(i-T) + v(v-T)))}}\right) \\
h_{L[x]}^2 &= \frac{2\Phi^{-1}(AUC)^2}{(v-i)^2 + \Phi^{-1}(AUC)^2(i(i-T) + v(v-T))}.
\end{aligned}$$

From these equations, we computed the proportion of genetic variance explained as the ratio of $h_{L[x]}^2$ to the total heritability of liability h_L^2, as derived in the reference above.

Bias-correction experiment

We identified two potential competing explanations that could account for the improved performance of the larger models. First, sparse regularization algorithms achieve sparsity through shrinkage of estimated SNP effect sizes. In order to achieve the levels of sparsity needed to attain the low false positive rate guaranteed by the model containing only genome-wide significant associations, the amount of shrinkage applied would need to be very large, potentially leading to underestimates of effect sizes for SNPs in the smallest models and thus artificially decreasing AUC. Second, the sparsity-inducing prior we used also had the effect of decreasing the number of correlated SNPs included in the models when the sparsity-inducing prior was strong. Such correlated SNPs, however, might be important if the variants included on the genotyping panel were not directly causal but rather tagging unobserved causal SNPs or risk haplotypes. For example, the H1 haplotype at the $MAPT$ locus spanning a large 900 KB region on chromosome 17 was tagged using four markers in the $E[FP] \leq 10$ model; the sparse classifier based on genome-wide significant associations, however, tagged the same region using only two markers. Here, based on the results in Table 4, one cannot exclude the possibility that the increased accuracy of the $E[FP] \leq 10$ might only be due to the inclusion of additional correlated SNPs in the $MAPT$ region.

To address these concerns, we ran a modified external validation experiment in which we compared versions of the genome-wide significant model against each of the four larger models without confounding due to shrinkage or the inclusion of linked SNPs. In particular, for each comparison, we first augmented the genome-wide significant model by including any SNPs in the larger model that were in LD ($r^2 \geq 0.1$) with the SNPs belonging to the genome-wide significant model. We then refit logistic regularization models on each set of SNPs, omitting the sparsity-inducing prior (although with a nominal fixed regularization term to ensure good convergence).

After these two corrections, the $E[FP] \leq 1$ and $E[FP] \leq 10$ models remain significantly better than their corresponding bias-corrected genome-wide significant models, with the best model achieving a bias-corrected AUC of 0.614 (see Supplementary Table S4). The $E[FP] \leq 100$ and $E[FP] \leq 1000$ models show a decrease in AUC. As confirmed by the large difference between expected and predicted AUCs for these two models, this decrease is evidence of overfitting as a consequence of the omission of the sparsity-inducing prior.

These results suggest that additional important loci are lurking beneath the genome-wide significance threshold within the $E[FP] \leq 1$ and $E[FP] \leq 10$ sets. Overfitting prevents us from reaching more concrete conclusions about the presence of additional true associations in the two larger sets.

References

1. Rosenbaum P (1991) A characterization of optimal designs for observational studies. J R Statist Soc B 53: 597–610.

2. Edmonds J, Karp RM (1972) Theoretical improvements in algorithmic efficiency for network flow problems. J ACM 19: 248–264.

3. Lee Y, Orlin JB (1993) QuickMatch: a very fast assignment for the assignment problem. MIT Sloan School Working Paper 3547–93.

4. Lunceford JK, Davidian M (2004) Stratification and weighting via the propensity score in estimation of causal treatment effects: a comparative study. Stat Med 23: 2937–2960.

5. Janes H, Pepe M (2009) Adjusting for covariate effects on classification accuracy using the covariate-adjusted receiver operating characteristic curve. Biometrika 96: 371–382.

6. DeLong E, DeLong D, Clarke-Pearson D (1988) Comparing the areas under two or more correlated receiver operating characteristic curves: a nonparametric approach. Biometrics 44: 837–845.

7. Bamber D (1975) The area above the ordinal dominance graph and the area below the receiver operating characteristic graph. Journal of Mathematical Psychology 12: 387–415.

8. Falconer DS (1965) The inheritance of liability to certain diseases, estimated from the incidence among relatives. Annals of Human Genetics 29: 51–76.

9. Wray NR, Yang J, Goddard ME, Visscher PM (2010) The genetic interpretation of area under the ROC curve in genomic profiling. PLoS Genet 6: e1000864.
